# Use of Genomics to Investigate Historical Importation of Shiga Toxin–Producing *Escherichia coli* Serogroup O26 and Nontoxigenic Variants into New Zealand

**DOI:** 10.3201/eid2503.180899

**Published:** 2019-03

**Authors:** A. Springer Browne, Patrick J. Biggs, David A. Wilkinson, Adrian L. Cookson, Anne C. Midwinter, Samuel J. Bloomfield, C. Reed Hranac, Lynn E. Rogers, Jonathan C. Marshall, Jackie Benschop, Helen Withers, Steve Hathaway, Tessy George, Patricia Jaros, Hamid Irshad, Yang Fong, Muriel Dufour, Naveena Karki, Taylor Winkleman, Nigel P. French

**Affiliations:** Massey University, Palmerston North, New Zealand (A.S. Browne, P.J. Biggs, D.A. Wilkinson, A.L. Cookson, A.C. Midwinter, S.J. Bloomfield, C.R. Hranac, L.E. Rogers, J.C. Marshall, J. Benschop, T. George, P. Jaros, H. Irshad, Y. Fong, T. Winkleman, N.P. French);; New Zealand Food Safety Science & Research Centre, Palmerston North (D.A. Wilkinson, N.P. French);; AgResearch Limited, Palmerston North (A.L. Cookson);; Ministry of Primary Industries, Wellington, New Zealand (H. Withers, S. Hathaway);; Institute of Environmental Science and Research, Upper Hutt, New Zealand (M. Dufour, N. Karki)

**Keywords:** Shiga-toxin–producing Escherichia coli, whole-genome sequencing, STEC, genetic evolution, cattle, zoonoses, bacteria, New Zealand, Japan, Escherichia coli, serogroup O26, enteric infections

## Abstract

Shiga toxin–producing *Escherichia coli* serogroup O26 is an important public health pathogen. Phylogenetic bacterial lineages in a country can be associated with the level and timing of international imports of live cattle, the main reservoir. We sequenced the genomes of 152 *E. coli* O26 isolates from New Zealand and compared them with 252 *E. coli* O26 genomes from 14 other countries. Gene variation among isolates from humans, animals, and food was strongly associated with country of origin and *stx* toxin profile but not isolation source. Time of origin estimates indicate serogroup O26 sequence type 21 was introduced at least 3 times into New Zealand from the 1920s to the 1980s, whereas nonvirulent O26 sequence type 29 strains were introduced during the early 2000s. New Zealand’s remarkably fewer introductions of Shiga toxin–producing *Escherichia coli* O26 compared with other countries (such as Japan) might be related to patterns of trade in live cattle.

Shiga toxin–producing *Escherichia coli* (STEC) is an important public health pathogen, capable of causing hemorrhagic diarrhea and life-threatening kidney failure, particularly in children (*1*). STEC is primarily transmitted through the fecal–oral route, and ruminants are important reservoirs of this zoonotic pathogen (*2*).

Initial research focused on STEC serotype O157:H7 as the main STEC pathogen involved in hemolytic uremic syndrome (*3*). However, STEC serogroup O26 has become an increasingly common cause of human disease. STEC O26 is the second most frequently detected serogroup causing STEC illness in New Zealand (*4*), the United States (*5*), and Europe (*6*).

Whole-genome sequencing (WGS) offers high-resolution identification of related bacterial isolates, while helping to direct source attribution investigations and interventions (*7*). The large amount of sequence data produced by such initiatives as GenomeTrakr (*8*) provides an opportunity to interpret the evolution and transmission of organisms across national boundaries.

New Zealand is a geographically isolated island nation that offers an opportunity to interpret the effects of importation and biosecurity measures on the control and transmission of zoonotic diseases (*9*). New Zealand has a relatively high incidence of notified human disease caused by STEC compared with other countries where the disease is notifiable; 11.9 STEC cases per 100,000 population were reported in New Zealand in 2017 (*10*), compared with 2.85 cases per 100,000 population in 2016 in the United States (*11*). A case–control study in New Zealand identified contact with animal manure and the presence of cattle in the local area as significant risk factors for human infection (*12*). After this case–control study, a New Zealand–wide cross-sectional study using a culture-independent test found STEC O26 in 7.2% of young dairy calves sampled (*13*).

Our objective was to compare genomes of *E. coli* serogroup O26 isolates from human clinical cases and cattle in New Zealand with genomes of bacterial isolates from non–New Zealand sources, examining the genetic diversity and population structure, evolution, time to most recent common ancestor (tMRCA), antimicrobial resistance, and virulence genes. These data can be used to infer the probable importation, transmission, and evolution of STEC O26, which can inform risk management decisions with regard to movement of reservoir animals, as well as potential interventions for public health. This research received Massey University Ethics approval (Notification No. 4000016530).

## Methods

### New Zealand Bacterial Isolates: Selection, DNA and Library Preparation, and Sequencing

We conducted random stratified selection, by year, region, farm, and source, of 152 serogroup O26 bacterial isolates from New Zealand human sources (32 isolates) and bovine sources (120 isolates) from 1985 to 2016. We previously analyzed a subset of 66 bovine isolates as part of a cross-sectional study of STEC prevalence on dairy farms (*13*). We obtained human isolates from the Institute of Environmental Science and Research (Wallaceville, New Zealand) and bovine isolates from the Hopkirk Research Institute at Massey University (Palmerston North, New Zealand). We extracted DNA from a single colony picked from Columbia Horse Blood Agar (Fort Richard Laboratories, http://www.fortrichard.com) using the QIAamp DNA MiniKit (QIAGEN, https://www.qiagen.com) and prepared sequencing libraries using the Nextera XT DNA Library Preparation Kit (Illumina, https://www.illumina.com). Prepared libraries were submitted to New Zealand Genomics Limited (University of Otago, https://www.otago.ac.nz/genomics/index.html), which performed sequencing using Illumina MiSeq 2 × 250 bp PE or Illumina HiSeq 2 × 125 bp PE v4.

Processed reads are publicly available on the National Center for Biotechnology Information Sequence Read Archive under BioProject ID PRJNA396667. Metadata are stored under BioSample accession nos. SAMN07430747–SAMN07430900 (Appendix 1 Table 1).

### Selection and Retrieval of Publicly Available *E. coli* Serogroup O26 Raw Sequence Data

To standardize the downstream genomics comparison pipeline, we included only bacterial isolates with raw sequencing data in this study. Isolate selection was stratified by country, year, and isolation source; we randomly selected up to 4 isolates from the same country, year, and isolation source. Raw beef samples were classified as bovine, whereas the food classification indicated nonmeat samples (e.g., spinach, flour). All potential sequences for this study were selected in December 2017, and 2 corresponding authors of publicly available assembled genomes (*14*,*15*) provided unpublished raw sequence data (S. Dellanoy, French Agency for Food, Environmental and Occupational Health & Safety, pers. comm., 2017 Jan 12; C. Gabrielsen, St. Olavs University Hospital, pers. comm., 2017 Jan 18). All 252 publicly available serogroup O26 sequences selected for this study are listed in Appendix 1 Table 2. ‬‬‬‬‬‬‬‬‬‬

### Assembly, Annotation, and Initial Analyses of WGS Data

We used the Nullarbor pipeline in the accurate mode (*16*) to evaluate, assemble, and annotate the WGS data and ECTyper to identify somatic (O) and flagellar (H) antigens (O:H serotype) (https://github.com/phac-nml/ecoli_serotyping). We identified virulence and resistance genes using ABRicate (https://github.com/tseemann/abricate), which bundles multiple databases for gene queries (Resfinder, CARD, ARG-ANNOT, NCBI BARRGD, NCBI, EcOH, PlasmidFinder, Ecoli_VF and VFDB). Identified attributes, metadata, virulence genes, and resistance genes for all genomes are provided in Appendix 2.

We performed pangenome analysis with the FindMyFriends package in the RStudio environment (*17*), which groups genes into orthologous clusters by implementing the cd-hit clustering algorithm (*18*), followed by a cluster refinement based on k-mer similarity. We examined pangenome composition using the HierarchicalSets package (*19*), estimating the similarity of isolates based on the number of shared (core) and characteristic (accessory/pan) genes. Gamma heterogeneity (the ratio of the number of core genes [intersect] to the number of pan genes [union]) was calculated for each group of genomes, and isolates are hierarchically clustered to minimize total heterogeneity, producing a dendrogram representation of genomic similarity.

We generated RaxML maximum-likelihood trees using a general time-reversible model from the concatenated alignment of all core genes outputted by FindMyFriends (*20*). Then, we created a dissimilarity matrix with the virulence gene output, based on the presence or absence of virulence genes between pairs of isolates, and used it to create neighbor-joining trees.

We evaluated the pangenome similarity matrix, as well as a dissimilarity matrix of the 192 virulence genes, with PERMANOVA (PRIMER-E; Quest Research Limited, https://www.primer-e.com) by using sequence type (ST), country, isolation source, and *stx* profile as independent factors. Phylogenetic figures were created using the iTOL (Interactive Tree of Life) software (*21*), and further amended using Inkscape open source software version 0.92.2 (https://inkscape.org).

### Single-Nucleotide Polymorphism Core Gene Alignment and tMRCA Analyses

We created a core gene alignment from the FindMyFriends package using DECIPHER (*22*). Two core alignments were performed for 2 STs: ST21 (345 isolates) and ST29 (48 isolates). Recombinant regions and identical isolates were removed using Gubbins 2.3.1 (*23*), resulting in 344 ST21 isolates and 48 ST29 isolates in the final analysis.

We determined the tMRCA using BEAST2 (*24*). The temporal signal was evaluated with BactDating (*25*) and found to be significant for both ST21 and ST29 data. Model evaluation of a combination of substitution, clock, and population models was performed using a method-of-moments estimator (*26*), and evaluation of log files using Tracer version 1.6.1 (http://tree.bio.ed.ac.uk/software/tracer/) led to a preferred model selection with the lowest AICM (Akaike’s information criterion for Markov chain Monte Carlo) estimates and consistent tracer line. General time-reversible substitution models were used to estimate tMRCAs with a coalescent extended Bayesian skyline model and relaxed molecular clock (*27*). tMRCA analysis was calibrated by tip dates (ST21, 1947–2017; ST29, 1952–2017); decimal dates were rounded to the middle of the month or year if an exact date was not available within the month or year. Effective sample size exceeded 100 for all models evaluated. Maximum-clade credibility trees were created using TreeAnnotator version 2.4.7 with a 10% burn-in (*24*). We determined the substitution rate for each ST, multiplying the substitution rate estimated by BEAST2 by the number of analyzed single-nucleotide polymorphisms (SNPs) and dividing the product by the mean genome size of the isolates analyzed.

### Cattle Importation Data

New Zealand cattle importation data were combined from a previous publication of historical importations of cattle into New Zealand (*28*) and Food and Agriculture Organization data from 1961 to 2013 (*29*). Live cattle imports into Japan from 1961 to 2013 were obtained from Food and Agriculture Organization data to enable us to compare them with New Zealand live cattle imports (*29*).

## Results

Most genomes were obtained from New Zealand (152 genomes), Japan (94 genomes), and the United States (79 genomes) (Table 1). Most isolates were ST21 (345 isolates) and ST29 (48 isolates); multiple *stx* gene profiles were represented (*stx1*, *stx2*, *stx1*, *stx2*, and no *stx*); and the source of isolates fell into 4 groups: human, bovine, food, and other animal.

**Table 1:** Summary of 404 *Escherichia coli* serogroup O26 isolates in an investigation of the bacterium’s historical importation into New Zealand

Country	*stx* Profile					Sequence type				Source			
	*stx1*	*stx2*	*stx1* and *stx2*	No *stx*		ST21	ST29	Other		Human	Bovine	Food	Other animal
Australia, n = 1	1	0	0	0		1	0	0		1	0	0	0
Belgium, n = 24	20	1	2	1		20	1	3		16	8	0	0
Continental Europe,* n = 21	3	13	2	3		6	13	2		19	2	0	0
Japan, n = 94	70	8	11	5		88	5	1		77	16	0	1
New Zealand, n = 152	104	0	0	48		136	16	0		32	120	0	0
Other North America,† n = 4	3	0	0	1		3	0	1		2	1	1	0
United Kingdom, n = 29	10	7	8	4		25	3	1		28	1	0	0
United States, n = 79	60	9	5	5		66	10	3		45	27	4	3
Total, n = 404	271	38	28	67		345	48	11		220	174	5	5

*Denmark, n = 1; France, n = 9; Germany, n = 6; Italy, n = 1; Norway, n = 2; Poland, n = 1; Switzerland, n = 1. †Canada, n = 3; Mexico, n = 1.

### Evolutionary Dynamics of *E. coli* Serogroup O26

**Figure 1:**
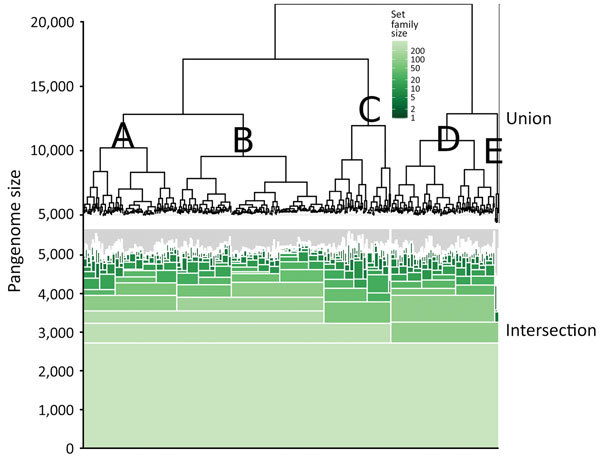
Hierarchical set analysis of 404 *Escherichia coli* serogroup O26 isolates in investigation of historical importation of Shiga toxin–producing *E. coli* serogroup O26 and nontoxigenic variants into New Zealand, with a hierarchical set RaxML pangenome tree (top of figure) and shared gene groups visualized in green (bottom of figure). This figure illustrates shared gene groups after pangenome analysis. The union portion represents the pangenome relatedness between bacterial isolates. A–E indicate clades.

**Figure 2:**
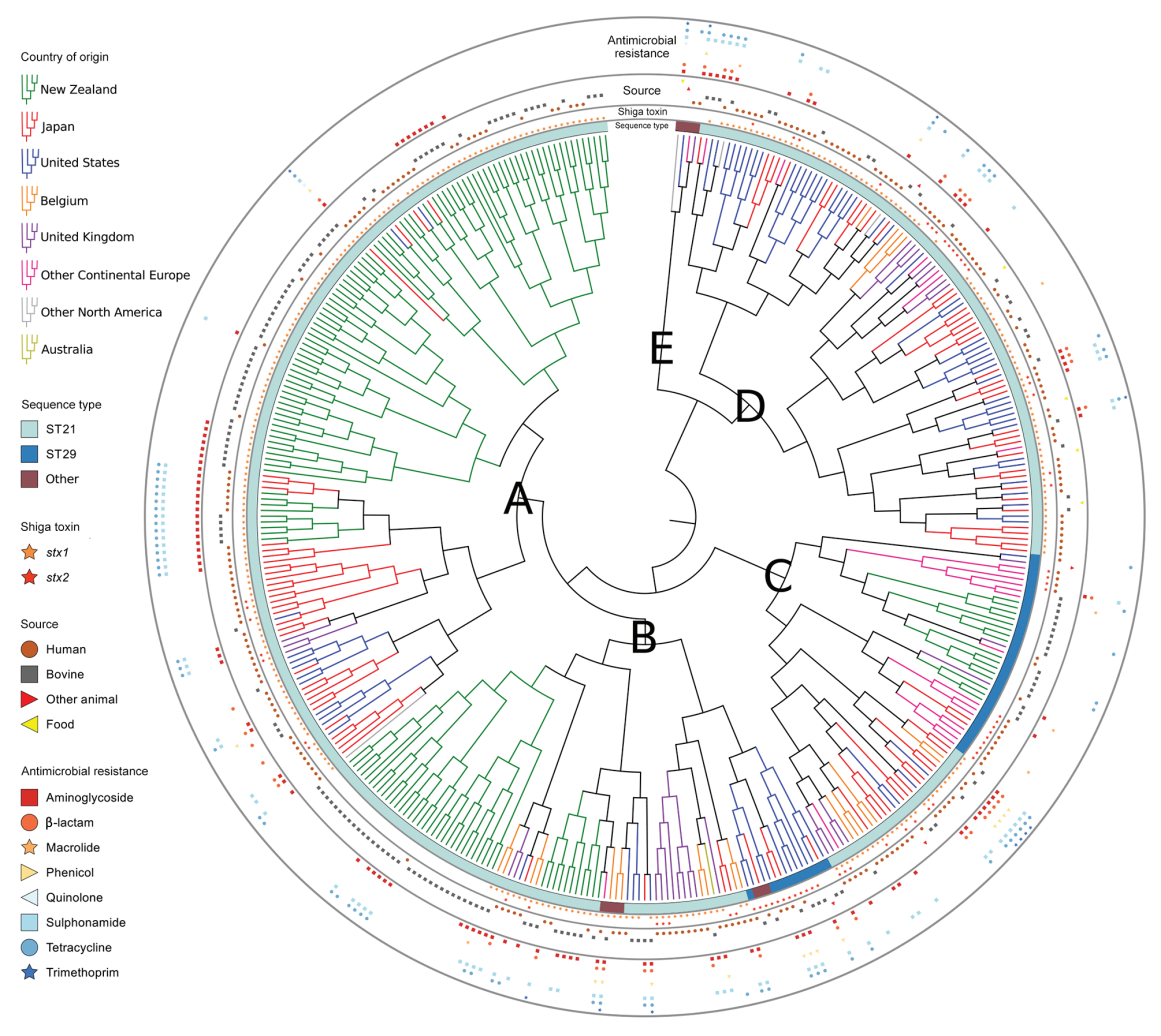
Hierarchical set RaxML tree of pangenome elements of 404 *Escherichia coli* serogroup O26 isolates in investigation of historical importation of Shiga toxin–producing *E.* serogroup O26 and nontoxigenic variants into New Zealand. A–E indicate clades, which are annotated. ST, sequence type.

Gene clustering analyses identified 2,718 core genes, 8,904 accessory genes, and 9,777 singleton genes, for a pangenome size of 21,399 genes. The pangenome was open and had a Heaps’ Law coefficient of 0.35. Hierarchical clustering based on pangenome composition enabled the definition of 5 independent classes of isolates (A–E) for comparative purposes; group E contained non-O26:H11 strains (Figure 1); clades A–D each share ≈4,000 core genes. A pangenome hierarchical set tree was annotated with country, ST, isolation source, and antimicrobial resistance gene class (Figure 2), with a real branch length figure available (Appendix 1 Figure 1). Multiple strains have circulated globally and are present in many countries.

PERMANOVA analysis for the pangenome and virulence genes revealed that gene variation among isolates was mostly explained by ST (pangenome, 33%; virulence genes, 84%), country of origin (pangenome, 18%; virulence genes, 2%), and *stx* profile (pangenome, 6%; virulence genes, 6%). Isolation source was not a significant factor (Table 2).

**Table 2:** PERMANOVA analysis of *Escherichia coli* serogroup O26 pangenome genes and virulence genes in an investigation of the bacterium’s historical importation into New Zealand*

Dataset, variable, no. genes	df	p value	Component of variation, %
Pangenome, n = 21,399			
Sequence type	5	0.0001	33
Country	14	0.0001	18
Isolation source	3	0.358	<0.01
*stx* profile	3	0.0001	6
Virulence genes, n = 192			
Sequence type	5	0.0001	83.7
Country	14	0.01	1.9
Isolation source	3	0.07	0.3
*stx* profile	3	0.0001	6.2

*This method determines whether the variation of the dataset is significantly associated with a particular variable. Residual variation: pangenome (43.0%), virulence genes (7.8%).

### Pathogenicity and Antimicrobial Resistance of *E. coli* O26

**Figure 3:**
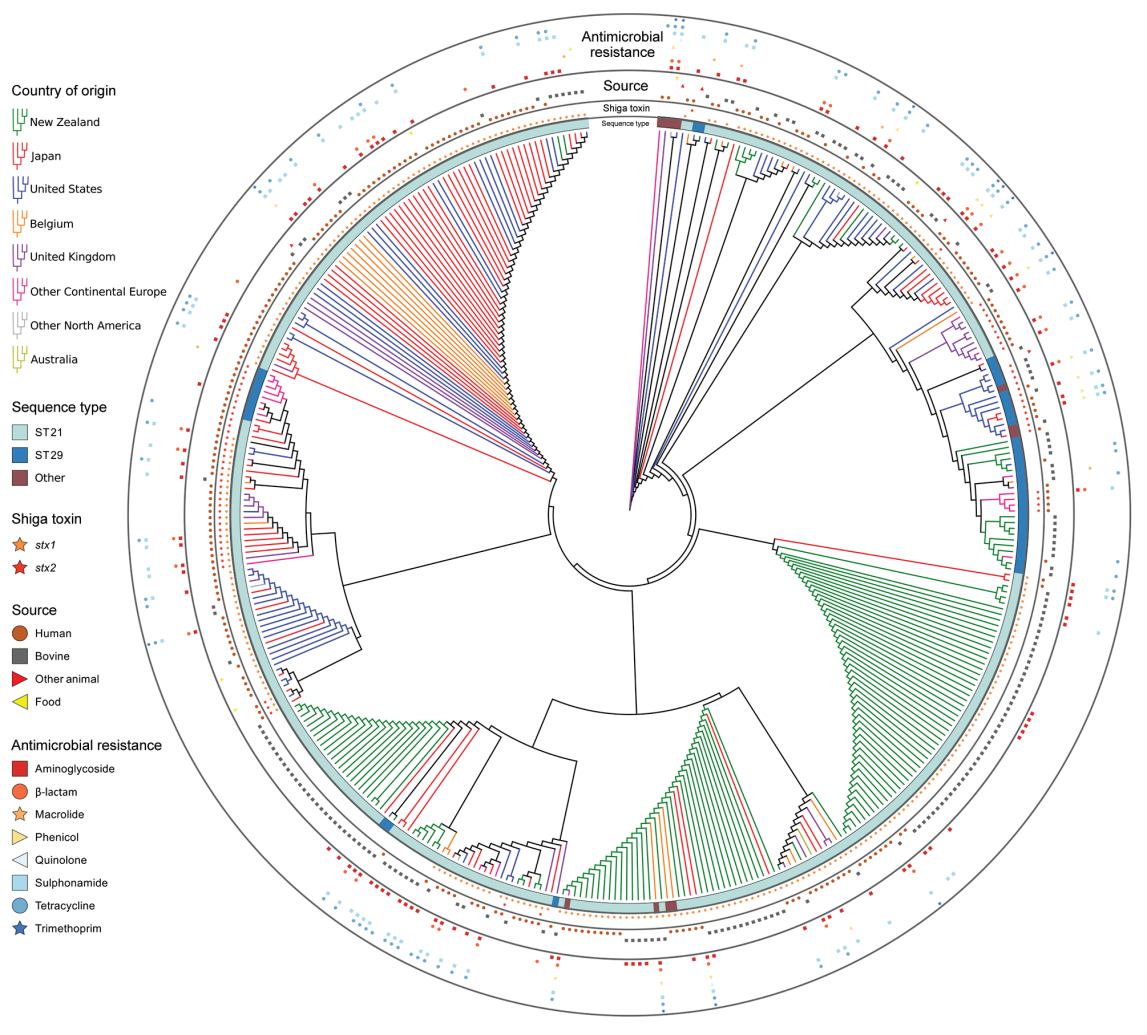
Neighbor-joining tree of 192 virulence genes of 404 *Escherichia coli* serogroup O26 isolates in investigation of historical importation of Shiga toxin–producing *E. coli* serogroup O26 and nontoxigenic variants into New Zealand. Branch lengths are ignored to better illustrate the country of origin of each isolate; therefore, closely spaced trellis-like branches have identical virulence profiles. ST, sequence type.

A neighbor-joining tree based on a distance matrix of the presence and absence of virulence genes detected (n = 192) (Figure 3) shows that a large number of New Zealand isolates had identical virulence profiles from human and bovine sources; a large clade from Japan, the United States, and Belgium also has identical profiles. We compiled a real branch length figure (Appendix 1 Figure 2), the name and function of all 192 detected virulence genes (Appendix 1 Table 3), and the virulence genes detected for each genome (Appendix 2).

We detected resistance genes for 8 classes of antimicrobial drugs (Table 3; Appendix 2 Table). Resistance genes were detected in 252 (62.4%) bacterial isolates.

**Table 3:** Detection of antimicrobial resistance genes of 404 *Escherichia coli* serogroup O26 isolates in an investigation of the bacterium’s historical importation into New Zealand*

Factor evaluated	Antimicrobial resistance, %							
	Aminoglycoside	β-lactam	Macrolide	Phenicol	Quinolone	Sulphonamide	Tetracycline	Trimethoprim
Country								
Australia, n = 1	100	0	0	100	0	100	0	0
Belgium, n = 24	67	25	4	17	0	67	42	17
Continental Europe, n = 21	29	14	10	5	0	24	14	0
Japan, n = 94	32	13	1	6	1	32	24	3
New Zealand, n = 152	26	1	1	0	0	12	12	1
Other North America, n = 4	50	25	25	0	25	50	100	0
United Kingdom, n = 29	24	21	0	4	0	29	25	4
United States, n = 79	13	13	0	4	0	4	14	3
Source*								
Human, n = 220	28	12	<1%	4	<1%	27	21	3
Bovine, n = 175	27	6	3	4	0	16	15	2
Food, n = 5	20	20	0	0	20	20	20	20
Total isolates, n = 404	30	10	2	4	1	23	19	3

*No antimicrobial resistance genes were detected in other animals (n = 5).

### tMRCA Analysis and Inferred Global Importation and Transmission of *E. coli* O26

**Figure 4:**
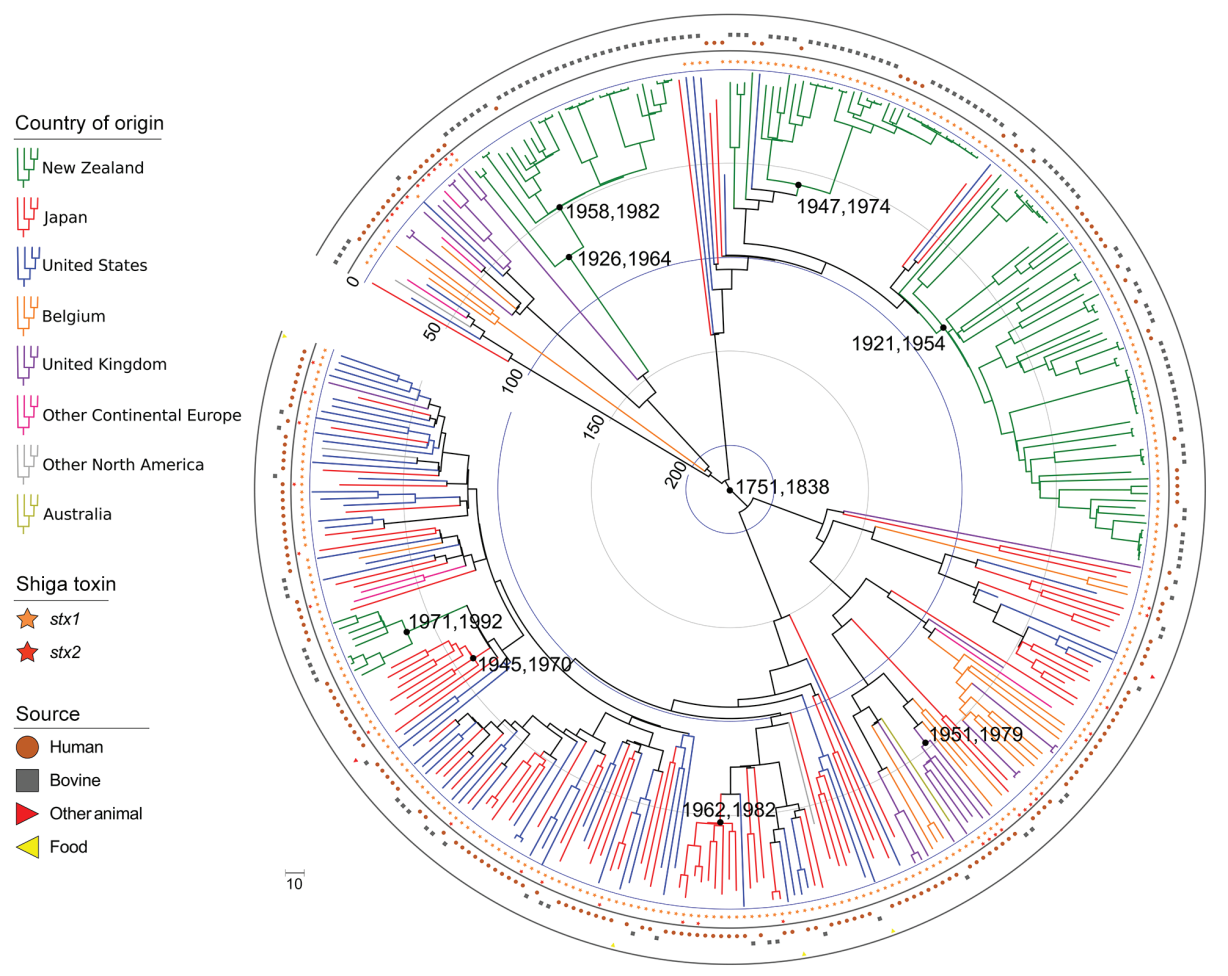
Maximum clade credibility tree of time of most recent common ancestor analysis of 344 *Escherichia coli* serogroup O26 sequence type 21 isolates in investigation of historical importation of Shiga toxin–producing *E. coli* serogroup O26 and nontoxigenic variants into New Zealand. Key convergence dates are annotated with 95% highest posterior density intervals, and the concentric circles indicate earlier time periods (blue, 100 years; gray, 50 years) from the age of the newest isolate (2017.5 in decimal years).

**Figure 5:**
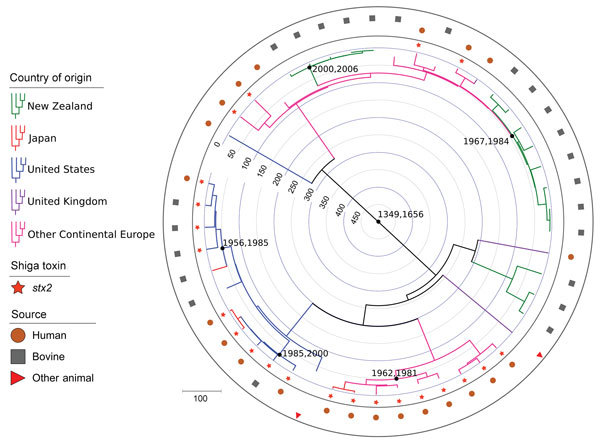
Maximum clade credibility tree of time of most recent common ancestor analysis of 48 *Escherichia coli* serogroup O26 sequence type 29 isolates in investigation of historical importation of Shiga toxin–producing *E. coli* serogroup O26 and nontoxigenic variants into New Zealand. Key convergence dates are annotated with 95% highest posterior density intervals, and concentric circles indicate prior time periods (blue, 100 years; gray, 50 years) from the age of the newest isolate (2017.0411 in decimal years).

A core gene alignment of the 344 serogroup O26 ST21 isolates generated 9,702 SNPs, and the 48 ST29 isolates generated 4,686 SNPs. In the tMRCA estimates for ST21 isolates (Figure 4) and ST29 isolates (Figure 5), important convergence dates were annotated with a 95% highest posterior density (HPD) interval (Appendix 1 Figures 3, 4). The calculated substitution rate for ST-21 was 1.4 × 10^−7^ substitutions/site/year (95% CI 1.1–1.7 × 10^−7^ substitutions/site/year), and the substitution rate for ST29 isolates was 3.2 × 10^−7^ substitutions/site/year (95% CI 2.3–3.9 × 10^−7^ substitutions/site/year).

Four New Zealand ST21 monophyletic clades indicate tMRCA estimates from the 1920s through the 1990s (Figure 4). Individual New Zealand monophyletic clades show evidence of importation from Europe (95% HPD interval 1958–1982) and more recently from the United States (95% HPD interval 1971–1992). Paraphyletic clades are visible from European sources, particularly from US and Japan isolates, which create a panmictic community, indicating frequent transmission between these countries. Two New Zealand ST29 monophyletic clades show tMRCA estimates from the late 1960s to the early 21st century (Figure 5). Japanese strains of 4 *stx2*-positive ST29 isolates appear to be closely related to strains from the United States and from Europe. Minimal evidence exists of transmission of New Zealand strains to the other countries evaluated in this study (Figures 4, 5).

**Figure 6:**
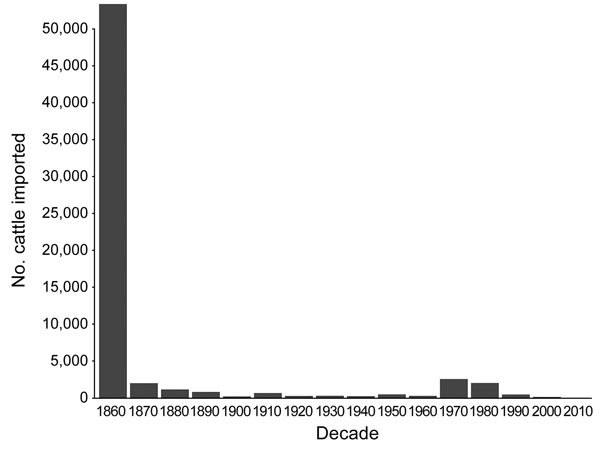
Historical importations of live cattle into New Zealand, 1860–2010.

**Figure 7:**
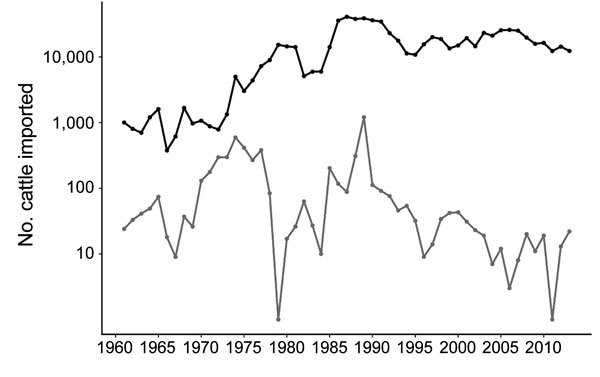
Comparison of live cattle imported (log_10_ scale) into New Zealand and Japan during 1961–2013. Japan, black; New Zealand, gray.

Most live cattle imported into New Zealand arrived during the 1860s (Figure 6), and importations increased during the 1950s–1990s. New Zealand imported fewer cattle than Japan for all years examined; since 1991, New Zealand has consistently imported <100 live cattle per year, whereas Japan has imported >10,000 per year (Figure 7).

## Discussion

We used WGS to compare New Zealand *E. coli* serogroup O26 bacteria with isolates from around the world. Our analyses demonstrated contrasting patterns of global panmixia and local isolation between different lineages of the O26 serogroup. Based on these patterns, we suggest that global O26 exchange most likely is linked to the import/export of live cattle; periods of between-country transmission occurred mainly during the 20th century. In New Zealand, several lineages have unique virulence profiles, each of which is likely to have been introduced at a single point in the recent past, and subsequently undergone local expansion and diversification. This country-specificity contrasts with other strains, notably from the United States and Japan, that appear to have been exchanged between geographic locations on multiple occasions over the same time.

Pangenome and virulence gene PERMANOVA analysis (Table 2) indicated that variation was best explained by multilocus subtype, country of origin, and *stx* profile. The lack of isolation source as a significant factor for pangenome or virulence gene analysis (Table 2) suggests that serogroup O26 isolates from humans, cattle, food, and other animals are not genetically differentiated and zoonotic transmission of this bacteria occurs frequently.

WGS analysis of STEC O157:H7 isolates in New Zealand showed findings similar to ours, where the within-country isolate diversity was unique and not related to the source of the isolate (*30*). As in our study, bovine and human New Zealand STEC O157:H7 isolates were closely related by genotyping, compared with isolates from the United States and Australia (*30*). Genes classified as virulent for humans are involved in the intestinal colonization of cattle (*31*,*32*); therefore, the contrasting virulence attributes associated with isolates from different countries might indicate separate niche adaptation and advantages for colonization of local host populations.

We observed that the *E. coli* O26 genome is open (Heaps’ Law coefficient 0.35), meaning that the suite of genes possessed by each isolate is highly variable. These genes will be picked up from their local environments. The Public Goods Hypothesis proposes that the horizontal exchange of widely available DNA sequences is the primary driver for local bacterial evolution (*33*). This horizontal exchange of DNA will influence virulence profiles and other phenotypic traits, such as antimicrobial resistance. Applying this hypothesis to the evolution of *E. coli* O26 implies continued adaptation of the bacterial strains in local environments as they further acquire and share genes, which ultimately could lead to the emergence of new pathogenic lineages of STEC.

Evidence of relatively recent acquisition of *stx2* virulence within STEC O26 ST29 is a cause of concern (*14*). Non-STEC ST29 strains are present in cattle in New Zealand, but at the time this article was written, no STEC O26 with the *stx2* virulence gene had been reported there. The emergence of highly pathogenic strains that harbor the *stx2* toxin gene has led to an increase in hemolytic uremic syndrome related to the O26 serogroup (*14*,*34*). Serogroup O26 ST29 *stx2* isolates also have been identified in Japan (*35*), and 2 separate clades of *stx2* ST29 isolates from Japan may have been imported from the United States and Europe (Figure 5). The lack of highly pathogenic ST29 *stx2* isolates in New Zealand might be due to few live cattle importations, as well as no major horizontal genetic transfer events of *stx2* to *E. coli* O26 in New Zealand. ‬‬‬‬‬‬‬‬‬‬‬‬‬‬‬‬‬‬

The resistance profiles form distinct combinations of resistance genes in isolates from particular countries (Figures 2, 3). Antimicrobial drugs are not usually prescribed for human STEC infections (*36*); however, selection pressure from antimicrobial drug use in livestock and humans with undiagnosed diarrheal illness may influence the evolution of resistance. Antimicrobial resistance from all human isolates was higher or equal to that of bovine isolates (Table 3), with the exception of that to macrolides.

The tMRCA of New Zealand *E. coli* O26 clades suggests several separate importations of strains that appear to coincide with cattle importation events (Figures 4–6). Phylogenetic analyses suggest certain New Zealand clades are more associated with specific geographic areas (e.g., United States or continental Europe), indicating that transmission pathways are likely to exist through live animal imports. The estimated substitution rates for ST21 and ST29 isolates in the present study are similar to previous estimates for serogroup O26 (2.8–4.3 × 10^−7^ substitutions/site/year) (*37*) and O157:H7 (*38*). The tMRCA of all ST21 (1751–1838) (Figure 4) is similar to tMRCA estimates of ≈213 years ago for a large ST21 clade evaluated by Ogura et al. (*37*).

We observed a remarkable difference between 2 island nations with the most bacterial isolates analyzed: New Zealand (n = 152) and Japan (n = 94). In the HierarchicalSets pangenome (Figure 2), virulence gene (Figure 3), and BEAST2 ST21 (Figure 4) analyses, New Zealand isolates show monophyletic clades, whereas Japan isolates are paraphyletic with US isolates. The historically larger number of live cattle importations into Japan than into New Zealand (Figure 7) might explain this difference. Japan was a major importer of US live cattle during the second half of the 20th century, until the detection of bovine spongiform encephalopathy led to a ban on all live cattle from the United States in 2003; current importations come from Australia (*39*,*40*). The relatively large number of imported live cattle in Japan could explain the different population structure of *E. coli* O26 in New Zealand and Japan.

Our dataset enables minimal interpretation of open border areas, such as the European Union or countries in the North American Free Trade Agreement (Mexico, Canada, and the United States), but our results from New Zealand suggest the introduction of serogroup O26 bacterial strains occurred during periods of intensive cattle importation. In cattle, STEC is a commensal bacterium and is shed intermittently (*41*); therefore, testing cattle before transportation is unrealistic. Our tMRCA and phylogenetic analyses suggest that minimal exchange of strains has occurred between countries in the 21st century; however, continued movement of cattle across international borders is likely to continue to influence the spread and genetic diversity of STEC around the world.

The results of our study are subject to several limitations. The quantity and diversity of *E. coli* O26 isolates from other countries were variable. More *E. coli* O26 isolates from Australia would have enabled us to better compare the effect of importation of cattle into New Zealand because Australia was the source of many historical cattle importations (*28*). Sequence data were more common from the past few years, and mostly human isolates were available. Although we randomly selected our New Zealand isolates from human and bovine isolates spanning >30 years from a diverse geographic range in New Zealand, some isolates were from the same farm (Appendix 1 Table 1), leading to a potential bias. Although non-STEC and STEC strains of the same serogroup are commonly of different lineages (*42*), our focus on a defined O surface antigen (O26) to classify bacterial isolates and evaluate evolutionary and phylogenetic relationships is consistent with other studies (*30*,*37*).

Our results suggest worldwide dissemination of multiple strains of ST21 and ST29 STEC and nontoxigenic serogroup O26 lineages occurred during the 20th century. Close genetic similarities between *E. coli* O26 isolated from multiple different sources indicates common transmission pathways among animals, food sources, and humans. The limited introductions of *E. coli* O26 strains into New Zealand are most likely linked to minimal importations of live cattle.

Further sequencing of historical isolates from multiple sources will improve evolutionary and epidemiologic studies. Full use of the genomic information of STEC will require a coordinated international approach to sequencing, data curation, analysis, and interpretation of those data (*43*). Although it is difficult to directly attribute transmission and emergence of STEC strains based on global historical events, interpreting evolutionary genomic data against economic and sociopolitical factors can help determine the drivers of pathogen emergence and dissemination, and inform future public health policy.

Appendix 1 Additional information about the use of genomics to investigate historical importation of Shiga toxin–producing *Escherichia coli* serogroup O26 and nontoxigenic variants into New Zealand.

Appendix 2 Additional information on 404 *Escherichia coli* O26 isolates used in investigation of historical importation of Shiga toxin–producing *E. coli* serogroup O26 and nontoxigenic variants into New Zealand.
