# Supplementary material for: Use of Genomics to Investigate Historical Importation of Shiga Toxin–Producing Escherichia coli Serogroup O26 and Nontoxigenic Variants into New Zealand
Source: Emerg Infect Dis. 2019 Mar;25(3):489–500. doi: 10.3201/eid2503.180899 (PMC6390770; doi:10.3201/eid2503.180899)
Supplement: Appendix 1 — Additional information about the use of genomics to investigate historical importation of Shiga toxin–producing Escherichia coli serogroup O26 and nontoxigenic variants into New Zealand. [file 18-0899-Techapp-s1.pdf]

Article DOI: https://doi.org/10.3201/eid2503.180899

Use of Genomics to Investigate Historical Importation of Shiga Toxin–Producing Escherichia coli Serogroup O26 and Nontoxigenic Variants into New Zealand

Appendix 1

Appendix 1 Table 1. Metadata for New Zealand isolates (BioProject PRJNA396667), including FarmID for isolates retrieved from the same farm

Sample_name	Accession	Instrument	Collection_date	Geo_loc_name	Isolation_source	Host_age	Serotype	Coverage	FarmID
100ST2	SAMN07430826	Illumina HiSeq 2500	1-Sep-08	New Zealand: Taranaki	Bovine	Calf	O26:H11	87x	
11ST	SAMN07430827	Illumina HiSeq 2501	19-Aug-08	New Zealand: Taranaki	Bovine	Calf	O26:H11	77x	
129ST2	SAMN07430779	Illumina HiSeq 2502	5-Sep-08	New Zealand: Manawatu-Wellington	Bovine	Calf	O26:H11	100x	
14ER2056	SAMN07430858	Illumina HiSeq 2503	12-Jul-14	New Zealand: Waikato	Human		O26:H11	112x	
14ER2240	SAMN07430859	Illumina HiSeq 2504	31-Jul-14	New Zealand: Waikato	Human		O26:H11	98x	
14ER3837	SAMN07430789	Illumina HiSeq 2505	13-Nov-14	New Zealand: Nelson Marlborough	Human		O26:H11	71x	
15ER0146	SAMN07430790	Illumina HiSeq 2506	7-Jan-15	New Zealand: Nelson Marlborough	Human		O26:H11	95x	
15ER0512	SAMN07430775	Illumina HiSeq 2507	3-Feb-15	New Zealand: Combined Auckland	Human		O26:H11	83x	
15ER0857	SAMN07430747	Illumina HiSeq 2508	27-Feb-15	New Zealand: Auckland	Human		O26:H11	80x	
15ER2552	SAMN07430776	Illumina HiSeq 2509	12-Aug-15	New Zealand: Combined Auckland	Human		O26:H11	118x	
15ER2837	SAMN07430748	Illumina HiSeq 2510	28-Aug-15	New Zealand: Auckland	Human		O26:H11	84x	
15ER3334	SAMN07430754	Illumina HiSeq 2511	26-Sep-15	New Zealand: Canterbury	Human		O26:H11	84x	
15ER3567	SAMN07430791	Illumina HiSeq 2512	13-Oct-15	New Zealand: Nelson Marlborough	Human		O26:H11	104x	
15ER3804	SAMN07430900	Illumina HiSeq 2513	1-Nov-15	New Zealand: Auckland	Human		O26:H11	94x	
15ER4241	SAMN07430749	Illumina HiSeq 2514	7-Dec-15	New Zealand: Auckland	Human		O26:H11	93x	
16ER0243	SAMN07430795	Illumina HiSeq 2515	14-Jan-16	New Zealand: Northland	Human		O26:H11	90x	
16ER0248	SAMN07430792	Illumina HiSeq 2516	13-Jan-16	New Zealand: Nelson Marlborough	Human		O26:H11	77x	
16ER0520	SAMN07430777	Illumina HiSeq 2517	4-Feb-16	New Zealand: Combined Auckland	Human		O26:H11	94x	
16ER0672	SAMN07430750	Illumina HiSeq 2518	2016	New Zealand: Auckland	Human		O26:H11	99x	
16ER0946	SAMN07430800	Illumina HiSeq 2519	4-Mar-16	New Zealand: Southland	Human		O26:H11	100x	
16ER1209	SAMN07430796	Illumina HiSeq 2520	23-Mar-16	New Zealand: Northland	Human		O26:H11	103x	

Sample_name	Accession	Instrument	Collection_date	Geo_loc_name	Isolation_source	Host_age	Serotype	Coverage	FarmID
16ER1432	SAMN07430751	Illumina HiSeq 2521	9-Apr-16	New Zealand: Auckland	Human		O26:H11	105x	
16ER1646	SAMN07430778	Illumina HiSeq 2522	30-Apr-16	New Zealand: Combined Auckland	Human		O26:H11	79x	
16ER1892	SAMN07430860	Illumina HiSeq 2523	20-May-16	New Zealand: Waikato	Human		O26:H11	91x	
16ER1973	SAMN07430793	Illumina HiSeq 2524	26-May-16	New Zealand: Nelson Marlborough	Human		O26:H11	109x	
16ER2139	SAMN07430861	Illumina HiSeq 2525	18-Jun-16	New Zealand: Waikato	Human		O26:H11	75x	
191ST2	SAMN07430828	Illumina HiSeq 2526	18-Sep-08	New Zealand: Taranaki	Bovine	Calf	O26:H11	104x	
22ST2	SAMN07430780	Illumina HiSeq 2527	19-Aug-08	New Zealand: Manawatu-Wellington	Bovine	Calf	O26:H11	81x	
66ST1	SAMN07430829	Illumina HiSeq 2528	27-Aug-08	New Zealand: Taranaki	Bovine	Calf	O26:H11	104x	
A14a	SAMN07430755	Illumina MiSeq	17-Aug-09	New Zealand: Canterbury	Bovine	Calf	O26:H11	164x	
A17a	SAMN07430756	Illumina MiSeq	17-Aug-09	New Zealand: Canterbury	Bovine	Calf	O26:H11	117x	
a185c	SAMN07430757	Illumina HiSeq 2531	19-Jan-11	New Zealand: Canterbury	Bovine	Cow	O26:H11	230x	
a234d	SAMN07430758	Illumina HiSeq 2532	15-Jun-11	New Zealand: Canterbury	Bovine	Cow	O26:H11	120x	
A46a	SAMN07430759	Illumina HiSeq 2533	30-Jul-10	New Zealand: Canterbury	Bovine	Calf	O26:H11	94x	
A65a	SAMN07430760	Illumina HiSeq 2534	16-Aug-10	New Zealand: Canterbury	Bovine	Calf	O26:H11	81x	
A65b	SAMN07430761	Illumina HiSeq 2535	16-Aug-10	New Zealand: Canterbury	Bovine	Calf	O26:H11	104x	
A87b	SAMN07430762	Illumina HiSeq 2536	6-Sep-10	New Zealand: Canterbury	Bovine	Calf	O26:H11	104x	
AGR373	SAMN07430781	Illumina HiSeq 2537	12-Dec-02	New Zealand: Manawatu-Wellington	Bovine	Calf	O26:H11	110x	
AGR72	SAMN07430782	Illumina HiSeq 2538	21-Nov-02	New Zealand: Manawatu-Wellington	Bovine	Calf	O26:H11	111x	
e171b	SAMN07430831	Illumina HiSeq 2539	22-Feb-11	New Zealand: Taranaki	Bovine	Cow	O26:H11	110x	
e171c	SAMN07430832	Illumina HiSeq 2540	22-Feb-11	New Zealand: Taranaki	Bovine	Cow	O26:H11	100x	
e186a	SAMN07430833	Illumina HiSeq 2541	22-Mar-11	New Zealand: Taranaki	Bovine	Cow	O26:H11	102x	
E189b	SAMN07430834	Illumina HiSeq 2542	13-Sep-10	New Zealand: Taranaki	Bovine	Calf	O26:H11	73x	
e195b	SAMN07430835	Illumina HiSeq 2543	18-Apr-11	New Zealand: Taranaki	Bovine	Cow	O26:H11	114x	
e26a	SAMN07430836	Illumina HiSeq 2544	22-Dec-09	New Zealand: Taranaki	Bovine	Cow	O26:H11	68x	
e37c	SAMN07430837	Illumina HiSeq 2545	18-Jan-10	New Zealand: Taranaki	Bovine	Cow	O26:H11	89x	
E46a	SAMN07430838	Illumina HiSeq 2546	25-Aug-09	New Zealand: Taranaki	Bovine	Calf	O26:H11	105x	
E59b	SAMN07430839	Illumina HiSeq 2547	7-Sep-09	New Zealand: Taranaki	Bovine	Calf	O26:H11	97x	
ER005420	SAMN07430854	Illumina HiSeq 2548	2000	New Zealand: Unknown	Human		O26:H11	106x	
ER854674	SAMN07430855	Illumina HiSeq 2549	1985	New Zealand: Unknown	Human		O26:H11	100x	
ER985544	SAMN07430856	Illumina HiSeq 2550	1998	New Zealand: Unknown	Human		O26:H11	83x	
ERL023841	SAMN07430857	Illumina HiSeq 2551	2002	New Zealand: Unknown	Human		O26:H11	96x	
ERL071565	SAMN07430794	Illumina HiSeq 2552	10-May-07	New Zealand: Nelson Marlborough	Human		O26:H11	109x	
ERL093655	SAMN07430752	Illumina HiSeq 2553	31-Oct-09	New Zealand: Auckland	Human		O26:H11	87x	
ERL111686	SAMN07430763	Illumina HiSeq 2554	6-May-11	New Zealand: Canterbury	Human		O26:H11	78x	

Sample_name	Accession	Instrument	Collection_date	Geo_loc_name	Isolation_source	Host_age	Serotype	Coverage	FarmID
ERL121992	SAMN07430797	Illumina HiSeq 2555	20-Feb-12	New Zealand: Northland	Human		O26:H11	95x	
H108a	SAMN07430798	Illumina MiSeq	21-Aug-09	New Zealand: Northland	Bovine	Calf	O26:H11	145x	
H113a	SAMN07430799	Illumina MiSeq	21-Aug-09	New Zealand: Northland	Bovine	Calf	O26:H11	143x	
H132a	SAMN07430862	Illumina HiSeq 2558	21-Aug-09	New Zealand: Waikato	Bovine	Calf	O26:H11	75x	
H13ESR01843	SAMN07430753	Illumina HiSeq 2559	20-Mar-13	New Zealand: Auckland	Human		O26:H11	103x	
h148a	SAMN07430863	Illumina HiSeq 2560	21-Feb-11	New Zealand: Waikato	Bovine	Cow	O26:H11	86x	
h148b	SAMN07430864	Illumina HiSeq 2561	21-Feb-11	New Zealand: Waikato	Bovine	Cow	O26:H11	71x	
h199a	SAMN07430865	Illumina HiSeq 2562	14-Jun-11	New Zealand: Waikato	Bovine	Cow	O26:H11	75x	
h199c	SAMN07430866	Illumina HiSeq 2563	14-Jun-11	New Zealand: Waikato	Bovine	Cow	O26:H11	67x	
H31c	SAMN07430867	Illumina HiSeq 2564	28-Jul-09	New Zealand: Waikato	Bovine	Calf	O26:H11	84x	
Hide14d	SAMN07430868	Illumina HiSeq 2565	10-Aug-10	New Zealand: Waikato	Bovine	Calf	O26:H11	120x	
ND29	SAMN07430869	Illumina HiSeq 2566	6-Nov-09	New Zealand: Waikato	Bovine	Calf	O26:H11	129x	
ND3	SAMN07430870	Illumina HiSeq 2567	21-Oct-09	New Zealand: Waikato	Bovine	Calf	O26:H11	125x	
ND35	SAMN07430871	Illumina HiSeq 2568	12-Nov-09	New Zealand: Waikato	Bovine	Calf	O26:H11	107x	
ND62	SAMN07430872	Illumina HiSeq 2569	24-Nov-09	New Zealand: Waikato	Bovine	Calf	O26:H11	119x	
Pre15a	SAMN07430873	Illumina HiSeq 2570	26-Jul-10	New Zealand: Waikato	Bovine	Calf	O26:H11	113x	
Pre4d	SAMN07430874	Illumina HiSeq 2571	3-Aug-10	New Zealand: Waikato	Bovine	Calf	O26:H11	115x	
T11i	SAMN07430801	Illumina HiSeq 2572	1-Sep-09	New Zealand: Southland	Bovine	Calf	O26:H11	95x	
t126c	SAMN07430802	Illumina HiSeq 2573	21-Sep-10	New Zealand: Southland	Bovine	Cow	O26:H11	93x	
t128a	SAMN07430803	Illumina HiSeq 2574	21-Dec-10	New Zealand: Southland	Bovine	Cow	O26:H11	98x	
t173a	SAMN07430804	Illumina HiSeq 2575	20-Apr-11	New Zealand: Southland	Bovine	Cow	O26:H11	165x	
t25a	SAMN07430805	Illumina HiSeq 2576	17-Dec-09	New Zealand: Southland	Bovine	Cow	O26:H11	101x	
t25c	SAMN07430806	Illumina HiSeq 2577	17-Dec-09	New Zealand: Southland	Bovine	Cow	O26:H11	105x	
T27a	SAMN07430807	Illumina MiSeq	14-Sep-09	New Zealand: Southland	Bovine	Calf	O26:H11	142x	
T48b	SAMN07430808	Illumina HiSeq 2579	17-Aug-10	New Zealand: Southland	Bovine	Calf	O26:H11	109x	
T66c	SAMN07430809	Illumina HiSeq 2580	17-Aug-10	New Zealand: Southland	Bovine	Calf	O26:H11	125x	
VC1113e	SAMN07430840	Illumina HiSeq 2581	4-Sep-14	New Zealand: Taranaki	Bovine	Calf	O26:H11	73x	VCF75
VC1122f	SAMN07430841	Illumina MiSeq	4-Sep-14	New Zealand: Taranaki	Bovine	Calf	O26:H11	129x	VCF76
VC1125e	SAMN07430842	Illumina HiSeq 2583	4-Sep-14	New Zealand: Taranaki	Bovine	Calf	O26:H11	95x	VCF76
VC1139e	SAMN07430764	Illumina MiSeq	11-Sep-14	New Zealand: Canterbury	Bovine	Calf	O26:H11	114x	VCF77
VC1140e	SAMN07430765	Illumina HiSeq 2585	11-Sep-14	New Zealand: Canterbury	Bovine	Calf	O26:H11	90x	VCF77
VC1186e	SAMN07430766	Illumina HiSeq 2586	15-Sep-14	New Zealand: Canterbury	Bovine	Calf	O26:H11	81x	VCF80
VC1187e	SAMN07430767	Illumina HiSeq 2587	15-Sep-14	New Zealand: Canterbury	Bovine	Calf	O26:H11	125x	VCF80
VC1190e	SAMN07430768	Illumina HiSeq 2588	15-Sep-14	New Zealand: Canterbury	Bovine	Calf	O26:H11	81x	VCF81

Sample_name	Accession	Instrument	Collection_date	Geo_loc_name	Isolation_source	Host_age	Serotype	Coverage	FarmID
VC1195e	SAMN07430769	Illumina HiSeq 2589	15-Sep-14	New Zealand: Canterbury	Bovine	Calf	O26:H11	78x	VCF81
VC1196e	SAMN07430770	Illumina HiSeq 2590	15-Sep-14	New Zealand: Canterbury	Bovine	Calf	O26:H11	93x	VCF81
VC1202e	SAMN07430771	Illumina HiSeq 2591	15-Sep-14	New Zealand: Canterbury	Bovine	Calf	O26:H11	64x	VCF81
VC1309e	SAMN07430772	Illumina HiSeq 2592	17-Sep-14	New Zealand: Canterbury	Bovine	Calf	O26:H11	79x	VCF89
VC1310e	SAMN07430773	Illumina HiSeq 2593	17-Sep-14	New Zealand: Canterbury	Bovine	Calf	O26:H11	158x	VCF89
VC1311e	SAMN07430774	Illumina HiSeq 2594	17-Sep-14	New Zealand: Canterbury	Bovine	Calf	O26:H11	75x	VCF89
VC1362e	SAMN07430810	Illumina HiSeq 2595	22-Sep-14	New Zealand: Southland	Bovine	Calf	O26:H11	112x	VCF93
VC1366e	SAMN07430811	Illumina HiSeq 2596	22-Sep-14	New Zealand: Southland	Bovine	Calf	O26:H11	57x	VCF93
VC1367e	SAMN07430812	Illumina HiSeq 2597	22-Sep-14	New Zealand: Southland	Bovine	Calf	O26:H11	87x	VCF93
VC1394e	SAMN07430813	Illumina MiSeq	23-Sep-14	New Zealand: Southland	Bovine	Calf	O26:H11	97x	VCF95
VC1395e	SAMN07430814	Illumina HiSeq 2599	23-Sep-14	New Zealand: Southland	Bovine	Calf	O26:H11	45x	VCF95
VC1395f	SAMN07430815	Illumina HiSeq 2600	23-Sep-14	New Zealand: Southland	Bovine	Calf	O26:H11	102x	VCF95
VC1395g	SAMN07430816	Illumina HiSeq 2601	23-Sep-14	New Zealand: Southland	Bovine	Calf	O26:H11	71x	VCF95
VC1396e	SAMN07430817	Illumina HiSeq 2602	23-Sep-14	New Zealand: Southland	Bovine	Calf	O26:H11	111x	VCF95
VC1403e	SAMN07430818	Illumina HiSeq 2603	23-Sep-14	New Zealand: Southland	Bovine	Calf	O26:H11	143x	VCF95
VC1471e	SAMN07430819	Illumina MiSeq	24-Sep-14	New Zealand: Southland	Bovine	Calf	O26:H11	124x	VCF100
VC1471f	SAMN07430820	Illumina HiSeq 2605	24-Sep-14	New Zealand: Southland	Bovine	Calf	O26:H11	136x	VCF100
VC1471g	SAMN07430821	Illumina HiSeq 2606	24-Sep-14	New Zealand: Southland	Bovine	Calf	O26:H11	70x	VCF100
VC1471h	SAMN07430822	Illumina HiSeq 2607	24-Sep-14	New Zealand: Southland	Bovine	Calf	O26:H11	70x	VCF100
VC1473e	SAMN07430823	Illumina HiSeq 2608	24-Sep-14	New Zealand: Southland	Bovine	Calf	O26:H11	126x	VCF100
VC1474e	SAMN07430824	Illumina HiSeq 2609	24-Sep-14	New Zealand: Southland	Bovine	Calf	O26:H11	97x	VCF100
VC1486e	SAMN07430825	Illumina HiSeq 2610	24-Sep-14	New Zealand: Southland	Bovine	Calf	O26:H11	75x	VCF101
VC396e	SAMN07430875	Illumina HiSeq 2611	12-Aug-14	New Zealand: Waikato	Bovine	Calf	O26:H11	178x	VCF27
VC397e	SAMN07430876	Illumina HiSeq 2612	12-Aug-14	New Zealand: Waikato	Bovine	Calf	O26:H11	51x	VCF27
VC401e	SAMN07430877	Illumina HiSeq 2613	12-Aug-14	New Zealand: Waikato	Bovine	Calf	O26:H11	65x	VCF31
VC452e	SAMN07430878	Illumina HiSeq 2614	13-Aug-14	New Zealand: Waikato	Bovine	Calf	O26:H11	70x	VCF31
VC456e	SAMN07430879	Illumina HiSeq 2615	13-Aug-14	New Zealand: Waikato	Bovine	Calf	O26:H11	84x	VCF31

Sample_name	Accession	Instrument	Collection_date	Geo_loc_name	Isolation_source	Host_age	Serotype	Coverage	FarmID
VC459e	SAMN07430880	Illumina HiSeq 2616	13-Aug-14	New Zealand: Waikato	Bovine	Calf	O26:H11	75x	VCF31
VC473e	SAMN07430881	Illumina HiSeq 2617	13-Aug-14	New Zealand: Waikato	Bovine	Calf	O26:H11	71x	VCF32
VC474e	SAMN07430882	Illumina HiSeq 2618	13-Aug-14	New Zealand: Waikato	Bovine	Calf	O26:H11	88x	VCF32
VC474f	SAMN07430883	Illumina HiSeq 2619	13-Aug-14	New Zealand: Waikato	Bovine	Calf	O26:H11	69x	VCF32
VC474g	SAMN07430884	Illumina HiSeq 2620	13-Aug-14	New Zealand: Waikato	Bovine	Calf	O26:H11	65x	VCF32
VC474h	SAMN07430885	Illumina HiSeq 2621	13-Aug-14	New Zealand: Waikato	Bovine	Calf	O26:H11	75x	VCF32
VC476e	SAMN07430886	Illumina HiSeq 2622	13-Aug-14	New Zealand: Waikato	Bovine	Calf	O26:H11	88x	VCF32
VC479e	SAMN07430888	Illumina HiSeq 2623	13-Aug-14	New Zealand: Waikato	Bovine	Calf	O26:H11	61x	VCF32
VC545e	SAMN07430889	Illumina HiSeq 2624	18-Aug-14	New Zealand: Waikato	Bovine	Calf	O26:H11	91x	VCF35
VC547e	SAMN07430890	Illumina HiSeq 2625	18-Aug-14	New Zealand: Waikato	Bovine	Calf	O26:H11	68x	VCF35
VC550e	SAMN07430891	Illumina HiSeq 2626	18-Aug-14	New Zealand: Waikato	Bovine	Calf	O26:H11	105x	VCF35
VC554e	SAMN07430892	Illumina HiSeq 2627	18-Aug-14	New Zealand: Waikato	Bovine	Calf	O26:H11	104x	VCF35
VC555e	SAMN07430893	Illumina HiSeq 2628	18-Aug-14	New Zealand: Waikato	Bovine	Calf	O26:H11	77x	VCF35
VC833e	SAMN07430783	Illumina MiSeq	27-Aug-14	New Zealand: Manawatu-Wellington	Bovine	Calf	O26:H11	117x	VCF56
VC833f	SAMN07430784	Illumina HiSeq 2630	27-Aug-14	New Zealand: Manawatu-Wellington	Bovine	Calf	O26:H11	62x	VCF56
VC833g	SAMN07430785	Illumina HiSeq 2631	27-Aug-14	New Zealand: Manawatu-Wellington	Bovine	Calf	O26:H11	98x	VCF56
VC833h	SAMN07430786	Illumina HiSeq 2632	27-Aug-14	New Zealand: Manawatu-Wellington	Bovine	Calf	O26:H11	52x	VCF56
VC836e	SAMN07430787	Illumina HiSeq 2633	27-Aug-14	New Zealand: Manawatu-Wellington	Bovine	Calf	O26:H11	61x	VCF56
VC837e	SAMN07430788	Illumina HiSeq 2634	27-Aug-14	New Zealand: Manawatu-Wellington	Bovine	Calf	O26:H11	78x	VCF56
VC880e	SAMN07430843	Illumina HiSeq 2635	1-Sep-14	New Zealand: Taranaki	Bovine	Calf	O26:H11	54x	VCF59
VC932f	SAMN07430844	Illumina HiSeq 2636	1-Sep-14	New Zealand: Taranaki	Bovine	Calf	O26:H11	74x	VCF63
VC936e	SAMN07430845	Illumina HiSeq 2637	1-Sep-14	New Zealand: Taranaki	Bovine	Calf	O26:H11	97x	VCF63
VC940e	SAMN07430846	Illumina HiSeq 2638	1-Sep-14	New Zealand: Taranaki	Bovine	Calf	O26:H11	102x	VCF63
VC943e	SAMN07430847	Illumina MiSeq	2-Sep-14	New Zealand: Taranaki	Bovine	Calf	O26:H11	115x	VCF64
VC943f	SAMN07430848	Illumina HiSeq 2640	2-Sep-14	New Zealand: Taranaki	Bovine	Calf	O26:H11	84x	VCF64
VC943g	SAMN07430849	Illumina HiSeq 2641	2-Sep-14	New Zealand: Taranaki	Bovine	Calf	O26:H11	102x	VCF64
VC943h	SAMN07430850	Illumina HiSeq 2642	2-Sep-14	New Zealand: Taranaki	Bovine	Calf	O26:H11	64x	VCF64
VC946e	SAMN07430851	Illumina HiSeq 2643	2-Sep-14	New Zealand: Taranaki	Bovine	Calf	O26:H11	101x	VCF64
VC951e	SAMN07430852	Illumina HiSeq 2644	2-Sep-14	New Zealand: Taranaki	Bovine	Calf	O26:H11	54x	VCF64
VC955e	SAMN07430853	Illumina HiSeq 2645	2-Sep-14	New Zealand: Taranaki	Bovine	Calf	O26:H11	74x	VCF64
VL0828h	SAMN07430894	Illumina HiSeq 2646	20-Aug-15	New Zealand: Waikato	Bovine	Calf	O26:H11	94x	
VL0958f	SAMN07430895	Illumina HiSeq 2647	1-Sep-15	New Zealand: Waikato	Bovine	Calf	O26:H11	98x	
VL1058g	SAMN07430896	Illumina HiSeq 2648	7-Sep-15	New Zealand: Waikato	Bovine	Calf	O26:H11	145x	
VL1277e	SAMN07430897	Illumina HiSeq 2649	12-Jul-16	New Zealand: Waikato	Bovine	Calf	O26:H11	124x	
VL2020e	SAMN07430898	Illumina HiSeq 2650	9-Aug-16	New Zealand: Waikato	Bovine	Calf	O26:H11	112x	
VL2228e	SAMN07430899	Illumina HiSeq 2651	29-Aug-16	New Zealand: Waikato	Bovine	Calf	O26:H11	135x	

Appendix 1 Table 2. Bioproject, BioSample, SRA run number, country, source, and unique identifier for all public sequence data used in this study (n = 252)

BioProject	BioSample	SRA run	Country	Source	Current_study_ID
PRJNA230969	SAMN05607363	SRR5330941	Australia	Human	MOD1EC1684_Australia_human
PRJDB5571	SAMD00075768	DRR103425	Belgium	Bovine	357S89_Belgium_bovine
PRJDB5571	SAMD00075735	DRR103392	Belgium	Human	EH031_Belgium_human
PRJDB5571	SAMD00075761	DRR103418	Belgium	Bovine	631KH91_Belgium_bovine
PRJDB5571	SAMD00075766	DRR103423	Belgium	Human	EH182_Belgium_human
PRJDB5571	SAMD00075767	DRR103424	Belgium	Human	EH193_Belgium_human
PRJDB5571	SAMD00075736	DRR103393	Belgium	Human	EH322_Belgium_human
PRJDB5571	SAMD00075764	DRR103421	Belgium	Bovine	B44_Belgium_bovine
PRJDB5571	SAMD00075763	DRR103420	Belgium	Bovine	B43_Belgium_bovine
PRJDB5571	SAMD00075771	DRR103428	Belgium	Bovine	11KH263_Belgium_bovine
PRJDB5571	SAMD00075769	DRR103426	Belgium	Bovine	11KH63_Belgium_bovine
PRJDB5571	SAMD00075770	DRR103427	Belgium	Bovine	11KH245_Belgium_bovine
PRJDB5571	SAMD00075971	DRR103628	Belgium	Human	EH2035_Belgium_human
PRJDB5571	SAMD00075976	DRR103633	Belgium	Human	EH2083_Belgium_human
PRJDB5571	SAMD00075975	DRR103632	Belgium	Human	EH2075_Belgium_human
PRJDB5571	SAMD00075974	DRR103631	Belgium	Human	EH2068_Belgium_human
PRJDB5571	SAMD00075772	DRR103429	Belgium	Bovine	12KH23_Belgium_bovine
PRJDB5571	SAMD00075981	DRR103638	Belgium	Human	EH2208_Belgium_human
PRJDB5571	SAMD00075984	DRR103641	Belgium	Human	EH2244_Belgium_human
PRJDB5571	SAMD00075983	DRR103640	Belgium	Human	EH2219_Belgium_human
PRJDB5571	SAMD00075982	DRR103639	Belgium	Human	EH2209_Belgium_human
PRJDB5571	SAMD00075988	DRR103645	Belgium	Human	EH2258_Belgium_human
PRJDB5571	SAMD00075986	DRR103643	Belgium	Human	EH2252_Belgium_human
PRJDB5571	SAMD00075987	DRR103644	Belgium	Human	EH2257_Belgium_human
PRJDB5571	SAMD00075985	DRR103642	Belgium	Human	EH2251_Belgium_human
PRJNA319494	SAMN05504941*	SRR6154941	Canada	Bovine	OLC0637_Canada_bovine
PRJNA309770	SAMN04420181*	SRR6061322	Germany	Human	126814_Germany_human
PRJNA301341	SAMN04254589	SRR3110022	Canada	Human	EC120246_Canada_human
PRJEB10700	SAMEA3529294	ERR1010233	Denmark	Human	AA044_Denmark_human
PRJDB5571	SAMD00075758	DRR103415	France	Human	99109_France_human
PRJDB5571	SAMD00075757	DRR103414	France	Human	02113_France_human
PRJDB5571	SAMD00075759	DRR103416	France	Human	03139_France_human
PRJNA230969	SAMN05605330	SRR5330864	Germany	Human	MOD1EC2814_Germany_human
PRJNA230969	SAMN05607379	SRR5330926	Germany	Human	MOD1EC1664_Germany_human
PRJDB5571	SAMD00075765	DRR103422	Italy	Bovine	ED80_Italy_bovine
PRJNA230969	SAMN06555271	SRR5336246	Mexico	Food	MOD1EC5336_Mexico_food
PRJEB23743	SAMEA104413463	ERR2210764	Poland	Human	10016_Poland_human
PRJDB5571	SAMD00075756	DRR103413	Switzerland	Human	TC6167_Switzerland_human
PRJDB5571	SAMD00075762	DRR103419	UK	Human	H19_UK_human
PRJNA419720	SAMN08095914	SRR6321366	UK	Human	2M8BS8_UK_human
PRJNA419720	SAMN08095913	SRR6321365	UK	Human	I20VK7_UK_human
PRJNA419720	SAMN08095930	SRR6321271	UK	Human	KLAV92_UK_human
PRJNA419720	SAMN08095938	SRR6321331	UK	Human	HKCVSH_UK_human
PRJEB4681	SAMEA2204500	ERR435109	UK	Human	ECO0283_UK_humansepsis
PRJNA315192	SAMN06030740	SRR5031110	UK	Bovine	211644_UK_bovine
PRJDB5571	SAMD00075752	DRR103409	USA	Human	TC6165_USA_human
PRJDB5571	SAMD00075754	DRR103411	USA	Bovine	TC6169_USA_bovine
PRJDB5571	SAMD00075747	DRR103404	USA	Bovine	TC3486_USA_bovine
PRJDB5571	SAMD00075748	DRR103405	USA	Bovine	TC3630_USA_bovine
PRJDB5571	SAMD00075749	DRR103406	USA	Bovine	TC3656_USA_bovine
PRJDB5571	SAMD00075751	DRR103408	USA	Bovine	TC4219_USA_bovine
PRJDB5571	SAMD00075753	DRR103410	USA	Human	TC6168_USA_human
PRJNA218110	SAMN04498710	SRR3178054	USA	Human	2009C3689_USA_human
PRJNA218110	SAMN04498712	SRR3178056	USA	Human	2009C3996_USA_human
PRJNA218110	SAMN04633589	SRR3371771	USA	Human	2009C4747_USA_human
PRJNA218110	SAMN04633622	SRR3371781	USA	Human	2010C3051_USA_human
PRJNA218110	SAMN04625574	SRR3360206	USA	Human	2010C3902_USA_human
PRJNA218110	SAMN04625585	SRR3360195	USA	Human	2010C4430_USA_human
PRJNA218110	SAMN04625563	SRR3360216	USA	Human	2011C3270_USA_human
PRJNA218110	SAMN04913811	SRR4113678	USA	Human	PNUSAE002149_USA_human
PRJNA218110	SAMN04913824	SRR4300141	USA	Human	PNUSAE002166_USA_human
PRJNA218110	SAMN04625466	SRR3360241	USA	Human	2011C3506_USA_human
PRJNA218110	SAMN04578418	SRR3290033	USA	Human	2012C3101_USA_human
PRJNA218110	SAMN04495854	SRR3171841	USA	Human	2012C3912_USA_human
PRJNA218110	SAMN04578419	SRR3290038	USA	Human	2012C3102_USA_human
PRJNA218110	SAMN04498549	SRR3178026	USA	Human	2012C4606_USA_human
PRJNA218110	SAMN04192188	SRR3040537	USA	Human	PNUSAE001578_USA_human

BioProject	BioSample	SRA run	Country	Source	Current_study_ID
PRJNA218110	SAMN03838116	SRR2481234	USA	Human	PNUSAE000885_USA_human
PRJNA218110	SAMN04227723	SRR3040532	USA	Human	PNUSAE001573_USA_human
PRJNA218110	SAMN04075848	SRR2417066	USA	Human	PNUSAE001154_USA_human
PRJNA218110	SAMN03272820	SRR1738019	USA	Human	PNUSAE000133_USA_human
PRJNA218110	SAMN03151532	SRR1635531	USA	Human	PNUSAE000002_USA_human
PRJNA218110	SAMN03840334	SRR2121025	USA	Human	PNUSAE000539_USA_human
PRJNA218110	SAMN04075513	SRR2415794	USA	Human	PNUSAE001379_USA_human
PRJNA218110	SAMN04075843	SRR2415808	USA	Human	PNUSAE001373_USA_human
PRJNA218110	SAMN03775204	SRR2481344	USA	Human	PNUSAE000779_USA_human
PRJNA218110	SAMN04500985	SRR3189440	USA	Human	PNUSAE002228_USA_human
PRJNA218110	SAMN04588711	SRR3371981	USA	Human	PNUSAE002615_USA_human
PRJNA218110	SAMN05209084	SRR3644551	USA	Human	PNUSAE003275_USA_human
PRJNA218110	SAMN05203326	SRR3644569	USA	Human	PNUSAE003211_USA_human
PRJNA218110	SAMN07373072	SRR5870554	USA	Human	PNUSAE008468_USA_human
PRJNA230969	SAMN05605272	SRR5330849	USA	Human	MOD1EC1750_USA_human
PRJNA230969	SAMN04902887	SRR3465501	USA	Other animal	MOD1EC6201_USA_other animal
PRJNA230969	SAMN05605257	SRR5330857	USA	Human	MOD1EC1919_USA_human
PRJNA230969	SAMN05591573	SRR5330824	USA	Human	MOD1EC550_USA_human
PRJNA268206	SAMN06256289	SRR5202193	USA	Bovine	FSIS1609416_USA_bovine
PRJNA268206	SAMN04908471	SRR3457631	USA	Bovine	FSIS1606391_USA_bovine
PRJNA268206	SAMN06127049	SRR5091629	USA	Bovine	FSIS1608854_USA_bovine
PRJNA268206	SAMN06127045	SRR5091628	USA	Bovine	FSIS1608722_USA_bovine
PRJNA268206	SAMN07237071	SRR5683240	USA	Bovine	FSIS1701668_USA_bovine
PRJNA268206	SAMN06700862	SRR5441623	USA	Bovine	FSIS1710186_USA_bovine
PRJNA268206	SAMN07987839	SRR6265848	USA	Bovine	FSIS11704781_USA_bovine
PRJNA268206	SAMN07774189	SRR6158105	USA	Bovine	FSIS21720313_USA_bovine
PRJNA218110	SAMN05294505	SRR3883019	USA	Human	PNUSAE003398_USA_human
PRJNA218110	SAMN02352904	SRR3213940	USA	Human	643464_USA_human
PRJNA218110	SAMN02352964	SRR3371784	USA	Human	2010C3472_USA_human
PRJNA218110	SAMN08129177	SRR6359280	USA	Human	PNUSAE011184_USA_human
PRJNA230969	SAMN05439479	SRR3988028	USA	Food	MOD1EC6029_USA_food
PRJNA230969	SAMN05605269	SRR5330852	USA	Human	MOD1EC1753_USA_human
PRJNA230969	SAMN05605332	SRR5185399	Germany	Human	MOD1EC2812_Germany_human
PRJNA230969	SAMN05605331	SRR5185402	Germany	Human	MOD1EC2813_Germany_human
PRJNA230969	SAMN05605263	SRR5185394	Germany	Bovine	MOD1EC1763_Germany_bovine
PRJNA230969	SAMN03743659	SRR2176280	Canada	Human	CFSAN033951_Canada_human
PRJDB5571	SAMD00075828	DRR103485	Japan	Human	M01_Japan_human
PRJDB5571	SAMD00075829	DRR103486	Japan	Human	M02_Japan_human
PRJDB5571	SAMD00075830	DRR103487	Japan	Human	M03_Japan_human
PRJDB5571	SAMD00075832	DRR103489	Japan	Human	M05_Japan_human
PRJDB5571	SAMD00075905	DRR103562	Japan	Human	O01_Japan_human
PRJDB5571	SAMD00075998	DRR103655	Japan	Human	T02_Japan_human
PRJDB5571	SAMD00075999	DRR103656	Japan	Human	T03_Japan_human
PRJDB5571	SAMD00075911	DRR103568	Japan	Human	O07_Japan_human
PRJDB5571	SAMD00075785	DRR103442	Japan	Human	F02_Japan_human
PRJDB5571	SAMD00076003	DRR103660	Japan	Human	T08_Japan_human
PRJDB5571	SAMD00075908	DRR103565	Japan	Human	O04_Japan_human
PRJDB5571	SAMD00075912	DRR103569	Japan	Human	O08_Japan_human
PRJDB5571	SAMD00075786	DRR103443	Japan	Human	F03_Japan_human
PRJDB5571	SAMD00076004	DRR103661	Japan	Human	T09_Japan_human
PRJDB5571	SAMD00076010	DRR103667	Japan	Human	T16_Japan_human
PRJDB5571	SAMD00075789	DRR103446	Japan	Human	F06_Japan_human
PRJDB5571	SAMD00075848	DRR103505	Japan	Human	M21_Japan_human
PRJDB5571	SAMD00075918	DRR103575	Japan	Human	O16_Japan_human
PRJDB5571	SAMD00076017	DRR103674	Japan	Human	T23_Japan_human
PRJDB5571	SAMD00075930	DRR103587	Japan	Human	O29_Japan_human
PRJDB5571	SAMD00075927	DRR103584	Japan	Human	O25_Japan_human
PRJDB5571	SAMD00075852	DRR103509	Japan	Human	M25_Japan_human
PRJDB5571	SAMD00075793	DRR103450	Japan	Human	F10_Japan_human
PRJDB5571	SAMD00075728	DRR103385	Japan	Bovine	Aki01_Japan_bovine
PRJDB5571	SAMD00075856	DRR103513	Japan	Human	M30_Japan_human
PRJDB5571	SAMD00076025	DRR103682	Japan	Human	T32_Japan_human
PRJDB5571	SAMD00075936	DRR103593	Japan	Human	O35_Japan_human
PRJDB5571	SAMD00076024	DRR103681	Japan	Human	T31_Japan_human
PRJDB5571	SAMD00076026	DRR103683	Japan	Human	T33_Japan_human
PRJDB5571	SAMD00076028	DRR103685	Japan	Human	T35_Japan_human
PRJDB5571	SAMD00075799	DRR103456	Japan	Human	F17_Japan_human
PRJDB5571	SAMD00076030	DRR103687	Japan	Human	T39_Japan_human
PRJDB5571	SAMD00076032	DRR103689	Japan	Human	T41_Japan_human

BioProject	BioSample	SRA run	Country	Source	Current_study_ID
PRJDB5571	SAMD00075857	DRR103514	Japan	Human	M31_Japan_human
PRJDB5571	SAMD00075947	DRR103604	Japan	Human	O46_Japan_human
PRJDB5571	SAMD00075859	DRR103516	Japan	Human	M33_Japan_human
PRJDB5571	SAMD00076034	DRR103691	Japan	Human	T43_Japan_human
PRJDB5571	SAMD00076036	DRR103693	Japan	Human	T45_Japan_human
PRJDB5571	SAMD00075869	DRR103526	Japan	Human	M43_Japan_human
PRJDB5571	SAMD00075867	DRR103524	Japan	Human	M41_Japan_human
PRJDB5571	SAMD00075875	DRR103532	Japan	Human	M53_Japan_human
PRJDB5571	SAMD00075877	DRR103534	Japan	Human	M56_Japan_human
PRJDB5571	SAMD00075874	DRR103531	Japan	Human	M48_Japan_human
PRJDB5571	SAMD00075881	DRR103538	Japan	Human	M64_Japan_human
PRJDB5571	SAMD00076063	DRR103720	Japan	Bovine	YB02_Japan_bovine
PRJDB5571	SAMD00076062	DRR103719	Japan	Bovine	YB01_Japan_bovine
PRJDB5571	SAMD00075882	DRR103539	Japan	Human	M65_Japan_human
PRJDB5571	SAMD00076040	DRR103697	Japan	Human	T49_Japan_human
PRJDB5571	SAMD00076044	DRR103701	Japan	Human	T53_Japan_human
PRJDB5571	SAMD00075887	DRR103544	Japan	Human	M73_Japan_human
PRJDB5571	SAMD00076047	DRR103704	Japan	Human	T59_Japan_human
PRJDB5571	SAMD00076049	DRR103706	Japan	Human	T61_Japan_human
PRJDB5571	SAMD00075891	DRR103548	Japan	Human	M79_Japan_human
PRJDB5571	SAMD00075892	DRR103549	Japan	Human	M80_Japan_human
PRJDB5136	SAMD00064344	DRR073024	Japan	Human	NIID070765_Japan_human
PRJDB5571	SAMD00076064	DRR103721	Japan	Bovine	YB03_Japan_bovine
PRJDB5571	SAMD00075955	DRR103612	Japan	Human	O55_Japan_human
PRJDB5571	SAMD00075956	DRR103613	Japan	Human	O56_Japan_human
PRJDB5136	SAMD00064355	DRR073035	Japan	Human	NIID080884_Japan_human
PRJDB5571	SAMD00076067	DRR103724	Japan	Bovine	YB13_Japan_bovine
PRJDB5571	SAMD00076065	DRR103722	Japan	Bovine	YB05_Japan_bovine
PRJDB5571	SAMD00075782	DRR103439	Japan	Bovine	BK13_Japan_bovine
PRJDB5571	SAMD00076066	DRR103723	Japan	Bovine	YB06_Japan_bovine
PRJDB5571	SAMD00075893	DRR103550	Japan	Human	M81_Japan_human
PRJDB5571	SAMD00076055	DRR103712	Japan	Human	T67_Japan_human
PRJDB5571	SAMD00075958	DRR103615	Japan	Human	O58_Japan_human
PRJDB5571	SAMD00075957	DRR103614	Japan	Human	O57_Japan_human
PRJDB5571	SAMD00075898	DRR103555	Japan	Human	M89_Japan_human
PRJDB5571	SAMD00075901	DRR103558	Japan	Human	M92_Japan_human
PRJDB5571	SAMD00075899	DRR103556	Japan	Human	M90_Japan_human
PRJDB5571	SAMD00075824	DRR103481	Japan	Human	F44_Japan_human
PRJDB5571	SAMD00075780	DRR103437	Japan	Bovine	BK10_Japan_bovine
PRJDB5571	SAMD00075776	DRR103433	Japan	Bovine	BK05_Japan_bovine
PRJDB5571	SAMD00075781	DRR103438	Japan	Bovine	BK11_Japan_bovine
PRJDB5571	SAMD00075773	DRR103430	Japan	Bovine	BK01_Japan_bovine
PRJDB5571	SAMD00075962	DRR103619	Japan	Human	O62_Japan_human
PRJDB5136	SAMD00064358	DRR073038	Japan	Human	NIID111609_Japan_human
PRJDB5571	SAMD00075964	DRR103621	Japan	Human	O64_Japan_human
PRJDB5571	SAMD00075902	DRR103559	Japan	Human	M93_Japan_human
PRJDB5136	SAMD00064338	DRR073018	Japan	Human	NIID122711_Japan_human
PRJDB5136	SAMD00064353	DRR073033	Japan	Human	NIID121840_Japan_human
PRJDB5136	SAMD00064351	DRR073031	Japan	Human	NIID122657_Japan_human
PRJDB5136	SAMD00064348	DRR073028	Japan	Human	NIID122147_Japan_human
PRJDB5571	SAMD00075989	DRR103646	Japan	Bovine	She01_Japan_bovine
PRJDB5571	SAMD00075733	DRR103390	Japan	Bovine	Aki06_Japan_bovine
PRJDB5571	SAMD00075729	DRR103386	Japan	Bovine	Aki02_Japan_bovine
PRJDB5571	SAMD00075991	DRR103648	Japan	Bovine	She03_Japan_bovine
PRJDB5136	SAMD00064341	DRR073021	Japan	Human	NIID132777_Japan_human
PRJDB5136	SAMD00064342	DRR073022	Japan	Human	NIID141423_Japan_human
PRJDB5136	SAMD00064354	DRR073034	Japan	Human	NIID132265_Japan_human
PRJDB5136	SAMD00064337	DRR073017	Japan	Human	NIID130549_Japan_human
PRJDB5571	SAMD00075734	DRR103391	Japan	Other animal	Aki07_Japan_other animal
PRJDB5136	SAMD00064350	DRR073030	Japan	Human	NIID141424_Japan_human
PRJDB5136	SAMD00064343	DRR073023	Japan	Human	NIID141425_Japan_human
PRJNA315192	SAMN04568167	SRR3241986	UK	Human	93279_UK_human
PRJNA315192	SAMN04568166	SRR3241985	UK	Human	93280_UK_human
PRJNA315192	SAMN04567831	SRR3240981	UK	Human	93304_UK_human
PRJNA315192	SAMN04554948	SRR3226393	UK	Human	224098_UK_human
PRJNA315192	SAMN05734323	SRR4181480	UK	Human	146047_UK_human
PRJNA315192	SAMN05733983	SRR4179768	UK	Human	143934_UK_human
PRJNA259827	SAMN03703971	SRR2035403	UK	Human	46009_UK_human
PRJNA259827	SAMN03703997	SRR2035432	UK	Human	18109_UK_human

BioProject	BioSample	SRA run	Country	Source	Current_study_ID
PRJNA259827	SAMN03703954	SRR2120750	UK	Human	25910_UK_human
PRJNA259827	SAMN03703951	SRR2120773	UK	Human	60510_UK_human
PRJNA259827	SAMN03703966	SRR2120768	UK	Human	46710_UK_human
PRJNA259827	SAMN03703953	SRR2120771	UK	Human	51911_UK_human
PRJNA259827	SAMN03703952	SRR2035364	UK	Human	48212_UK_human
PRJNA259827	SAMN03703969	SRR2035399	UK	Human	62612_UK_human
PRJNA259827	SAMN03703970	SRR2035402	UK	Human	68013_UK_human
PRJNA259827	SAMN03703965	SRR2035374	UK	Human	63713_UK_human
PRJNA259827	SAMN03703967	SRR2035376	UK	Human	67013_UK_human
PRJNA315192	SAMN04568149	SRR3241859	UK	Human	93285_UK_human
PRJNA315192	SAMN05170656	SRR3578565	UK	Human	129381_UK_human
PRJNA315192	SAMN05733904	SRR4176976	UK	Human	143493_UK_human
PRJNA315192	SAMN05734341	SRR4181536	UK	Human	154820_UK_human
PRJNA315192	SAMN05734380	SRR4181594	UK	Human	152422_UK_human
PRJNA218110	SAMN02991228	SRR2014841	USA	Human	2010C4800_USA_human
PRJNA218110	SAMN03838114	SRR2481236	USA	Human	PNUSAE000887_USA_human
PRJNA218110	SAMN03785431	SRR2550380	USA	Human	PNUSAE000800_USA_human
PRJNA268206	SAMN04909393	SRR3457938	USA	Bovine	FSIS1503454_USA_bovine
PRJNA268206	SAMN03921926	SRR2125829	USA	Bovine	FSIS1500874_USA_bovine
PRJNA268206	SAMN03922101	SRR2126001	USA	Bovine	FSIS1500781_USA_bovine
PRJNA268206	SAMN03921925	SRR2125821	USA	Bovine	FSIS1500873_USA_bovine
PRJNA268206	SAMN03921934	SRR2125835	USA	Bovine	FSIS1500928_USA_bovine
PRJNA268206	SAMN03921935	SRR2125824	USA	Bovine	FSIS1500948_USA_bovine
PRJNA268206	SAMN03940972	SRR2132070	USA	Bovine	FSIS1503255_USA_bovine
PRJNA268206	SAMN03940974	SRR2132075	USA	Bovine	FSIS1503257_USA_bovine
PRJNA268206	SAMN03940973	SRR2132074	USA	Bovine	FSIS1503256_USA_bovine
PRJNA268206	SAMN03216753	SRR1693303	USA	Bovine	FSIS1400371_USA_bovine
PRJNA268206	SAMN03921981	SRR2125877	USA	Bovine	FSIS1500641_USA_bovine
PRJNA268206	SAMN03921972	SRR2125881	USA	Bovine	FSIS1500632_USA_bovine
PRJNA268206	SAMN03922009	SRR2125932	USA	Bovine	FSIS1500489_USA_bovine
PRJNA232925	SAMN02566899	SRR1272859	USA	Bovine	ewgs1005_USA_bovine
PRJNA283914	SAMN05366684	SRR4011138	USA	Other animal	WAPHLECOA00006_USA_caprine
PRJNA275276	SAMN03340674	SO174†	Norway	Human	SO174_Norway_human
PRJNA275276	SAMN03340677	SO179†	Norway	Human	SO179_Norway_human
PRJNA284656	SAMN03704971	36493†	France	Human	36493_France_human
PRJNA284656	SAMN03704970	36293†	France	Human	36293_France_human
PRJNA284656	SAMN03704969	36348†	France	Human	36348_France_human
PRJNA284656	SAMN03704968	34870†	France	Human	34870_France_human
PRJNA284656	SAMN03704966	36708†	France	Human	36708_France_human
PRJNA284656	SAMN03704963	36084†	France	Human	36084_France_human
PRJNA218110	SAMN04498563	SRR3178040	USA	Human	2015C5206_USA_human
PRJNA218110	SAMN04498702	SRR3178046	USA	Human	2016C3018_USA_human
PRJNA312475	SAMN05414569	SRR3931242	USA	Food	CFSAN046724_USA_food
PRJNA312475	SAMN05414581	SRR3931256	USA	Food	CFSAN046736_USA_food
PRJNA230969	SAMN05425824	SRR3938671	USA	Food	FDA00010430_USA_food
PRJNA230969	SAMN05452918	SRR3987970	USA	Avian	MOD1EC5703_USA_avian
PRJNA230969	SAMN05605333	SRR4340534	USA	Human	MOD1EC2790_USA_human

*Origin of isolate found by contacting submitter and original source.
†Raw sequence data provided by submi.

Appendix 1 Table 3. Virulence genes (n = 192) identified in serogroup O26 isolates (n = 404), annotated with function of gene

Gene name	Function
acpXL	Acyl carrier protein
afaA	Major fimbrial subunit of aggregative adherence fimbria II
afaB-I	Chaperone protein
afaC-VII	Putative AfaC-VIII usher protein
afaD-VII	Invasin protein
afaE-VII	Adhesin
afaG-VII	Adhesin
agg3B	Putative invasin protein
aslA	Putative arylsulfatase
astA	Heat-stable enterotoxin 1
cesAB	Chaperone for EspA and EspB
cesD	Chaperone for EspD and EspB
cesD2	Chaperone for EspD
cesF	Chaperone CesF
cesL	Chaperone for SepL
cesT	Chaperone for Tir
cheY	Chemotaxis proteinotaxis protein
chuS	Putative heme/hemoglobin transport proteinme/hemoglobin transport protein
chuU	iron ABC transporter permease
chuV	ATP binding hydrophilic protein
chuW	Coproporphyrinogen III oxidase
chuY	Hypothetical protein
cif	Type III secreted effector
cnf1	cytotoxic necrotizing factor 1
csgA	Curlin major subunit
csgB	Minor curlin subunit precursor
csgC	Curli assembly proteinoagglutination protein
csgD	Operon transcriptional regulatory protein
csgE	Curli production assembly/transport protein
csgF	Curli production assembly/transport protein
csgG	Curli production assembly/transport protein
eae	Intimin
east1	EAST1
efa1	EHEC factor for adherence
entA	2,3-dihydroxybenzoate-2,3-dehydrogenase
entB	Isochorismatase
entC	Isochorismate synthase 1
entD	Phosphopantetheinyl transferase component of enterobactin synthase multienzyme complex
entE	2,3-dihydroxybenzoate-AMP ligase component of enterobactin synthase multienzyme complex
entF	Enterobactin synthase multienzyme complex component, ATP-dependent
entS	Enterobactin exporter
escC	Type III secretion system secretin
escD	Type III secretion system outer MS ring protein
escE	Chaperone for EscF
escF	Type III secretion system needle filament protein
escG	Chaperone for EscF
escI	Type III secretion system inner rod component
escJ	Type III secretion system inner MS ring protein
escL	Negative regulator
escN	ATPase
escO	Positive regulator
escP	Type III secretion system needle length regulator
escR	Type III secretion systemminor export apparatus protein
escS	Type III secretion systemminor export apparatus protein
escT	Type III secretion system minor export apparatus protein
escU	Type III secretion system export apparatus switch protein
escV	Type III secretion system major export apparatus protein
espA	Type III secretion system
espD	Type III secretion system
espF	Type III secretion system
espG	Type III secretion system
espH	Type III secretion system
espJ	Prophage-encoded type III secretion system effector
espK	ESX-1 type VII secretion system
espL1	serine protease autotransporters of Enterobacteriaceae (SPATE)
espL2	serine protease autotransporters of Enterobacteriaceae (SPATE)

Gene name	Function
espL4	serine protease autotransporters of Enterobacteriaceae (SPATE)
espM1	BfpT-regulated chaperone
espM2	Putative T3SS effector protein
espN	T3SS effector
espP	Extracellular serine protease plasmid-encoded
espR1	Putative type III secreted effector
espW	T3SS effector
espX1	Putative type III secreted effector
espX2	Putative type III secreted effector
espX4	Putative type III secreted effector
espX5	Putative type III secreted effector
espX6	Putative type III secreted effector
espX7/nleL	Putative type III secreted effector
espY1	Apoptosis/cell cycle regulation
espY2	T3SS effector-like protein
espY3	Putative type III secreted effector
espY4	Putative type III secreted effector
etgA	T3SS-associated peptidoglycan lytic enzyme
etpB	Two-partner secretion transporter
faeC	K88 fimbrial protein
faeD	Outer membrane usher protein
faeE	Acyl-coenzyme A dehydrogenase
faeF	K88 minor fimbrial protein
faeH	K88 minor fimbrial protein
faeI	K88 minor fimbrial protein
faeJ	K88 minor fimbrial protein
fdeC	Adhesin
fepA	Ferrienterobactin outer membrane transporter
fepB	Ferrienterobactin ABC transporter periplasmic binding protein
fepC	Ferrienterobactin ABC transporter ATPase
fepD	Ferrienterobactin ABC transporter permease
fepG	Iron-enterobactin ABC transporter permease
fes	Enterobactin/ferric enterobactin esterase
fimA	Type-1 fimbrial protein
fimB	Type 1 fimbriae Regulatory protein
fimC	Chaperone protein
fimD	Outer membrane usher protein
fimE	Type 1 fimbriae Regulatory protein
fimF	Type 1 fimbrial minor component
fimG	Type 1 fimbrial minor component
fimH	Type 1 fimbrial adhesin precursor
fimI	ype 1 pilus biosynthesis fimbrial protein
flgD	Flagellar basal body rod modification protein
flgG	Flagellar basal-body rod protein
flgH	Flagellar L-ring protein precursor
flhA	Flagellar biosynthesis protein
fliG	Flagellar motor protein
fliI	Flagellum-specific ATP synthase
fliM	Flagellar motor switch protein
fliN	Flagellar motor switch protein
fliP	Flagellar biosynthesis protein
fyuA	Yersiniabactin receptor FyuA
gspC	General secretion pathway protein C
gspD	General secretion pathway protein D
gspE	General secretion pathway protein E
gspF	General secretion pathway protein F
gspG	General secretion pathway protein G
gspH	General secretion pathway protein H
gspI	General secretion pathway protein I
gspJ	General secretion pathway protein J
gspK	General secretion pathway protein K
gspL	General secretion pathway protein L
gspM	General secretion pathway protein M
gtrA	Bactoprenol-linked glucose translocase/flippase
gtrB	Bactoprenol glucosyl transferase
hlyA	Hemolysin transport protein
hlyB	Hemolysin transport protein
hlyC	Hemolysin transport protein
hlyD	Hemolysin transport protein

Gene name	Function
iroB	Glucosyltransferase
iroC	ABC transporter
iroD	Siderophore esterase
iroE	Siderophore esterase
iroN	Enterobactin siderophore receptor protein
irp1	Yersiniabactin polyketide synthase HMWP1
irp2	Yersiniabactin non-ribosomal peptide synthetase HMWP2
iucA	Aerobactin siderophore biosynthesis protein
iucB	Aerobactin siderophore biosynthesis protein
iucC	Aerobactin siderophore biosynthesis protein
iucD	L-lysine 6-monooxygenase
iutA	Ferric aerobactin receptor precusor
map	Rho guanine exchange factor
nleA	Non-LEE-encoded effector
nleB1	Non-LEE encoded effector B
nleB2	Non-LEE encoded effector B
nleC	Non-LEE encoded effector C
nleD	Type III secretion system effector
nleE	T3SS secreted effector protein
nleF	Effector protein
nleG7	Non-LEE-encoded type III effector
nleH1	Non-LEE-encoded type III effector
nleH2	Non-LEE-encoded type III effector
ompA	Outer membrane protein
ospG	Type III secretion system effector kinase
paa	Outer membrane adhesin
sepD	Type III secretion system secretion switch protein
sepL	Type III secretion system secretion gatekeeper
sepQ/escQ	Type III secetion system C ring protein
sepZ/espZ	Type III secretion system effector
set1A	Toxin subunit
set1B	Toxin subunit
shuA	Outer membrane heme/hemoglobin receptor
shuT	Periplasmic binding protein
shuX	Shu locus protein
spaQ	Type III secretion system minor export apparatus protein
stcE	Metalloprotease
stx1B	Shiga toxin 1, subunit B
stx2A	Shiga toxin 2, subunit A
stx2B	Shiga toxin 2, subunit B
stx1A	Shiga toxin 1, subunit A
toxB	Toxin B
vat	Vacuolating autotransporter toxin
yagV/ecpE	E. coli common pilus chaperone
yagW/ecpD	Polymerized tip adhesin of ECP fibers
yagX/ecpC	E. coli common pilus usher
yagY/ecpB	E. coli common pilus chaperone
yagZ/ecpA	E. coli common pilus structural subunit
ybtA	Yersiniabactin transcriptional regulator
ybtE	YersiniabYersiniabactin biosynthesis salycil-AMP ligase
ybtP	Yersiniabactin ABC transporter ATP binding/permease protein
ybtQ	Yersiniabactin ABC transporter ATP binding/permease protein
ybtS	Yersiniabactin biosynthesis salicylate synthase
ybtT	Yersiniabactin biosynthesis thioesterase
ybtU	Yersiniabactin biosynthesis oxidoreductase
ybtX	Yersiniabactin-associated zinc MFS transporter
ykgK/ecpR	Regulator protein

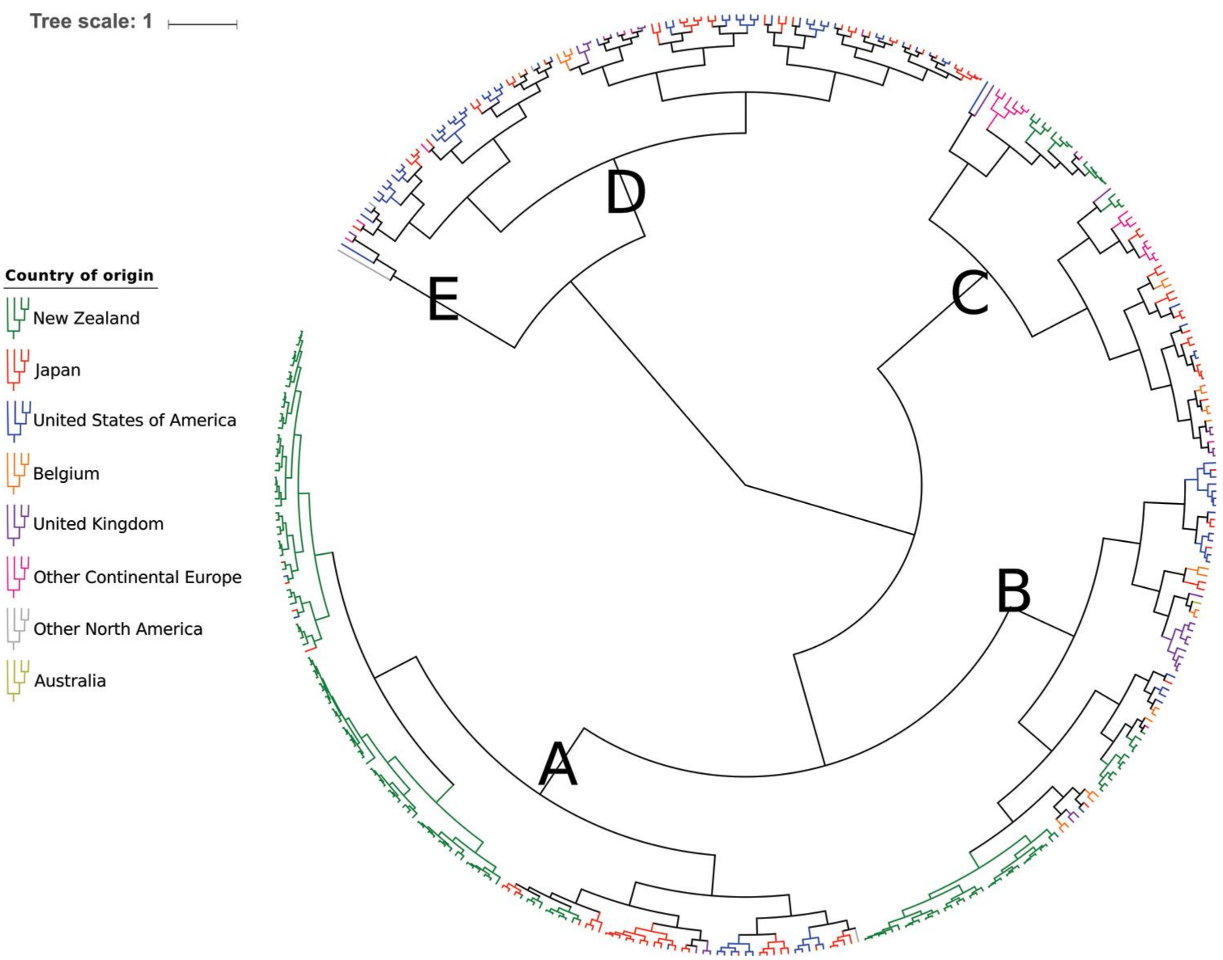

Appendix 1 Figure 1. RaxML maximum-likelihood hierarchical set tree of pangenome elements of Escherichia coli serogroup O26 isolates (n = 404), with real branch lengths and annotated by country.

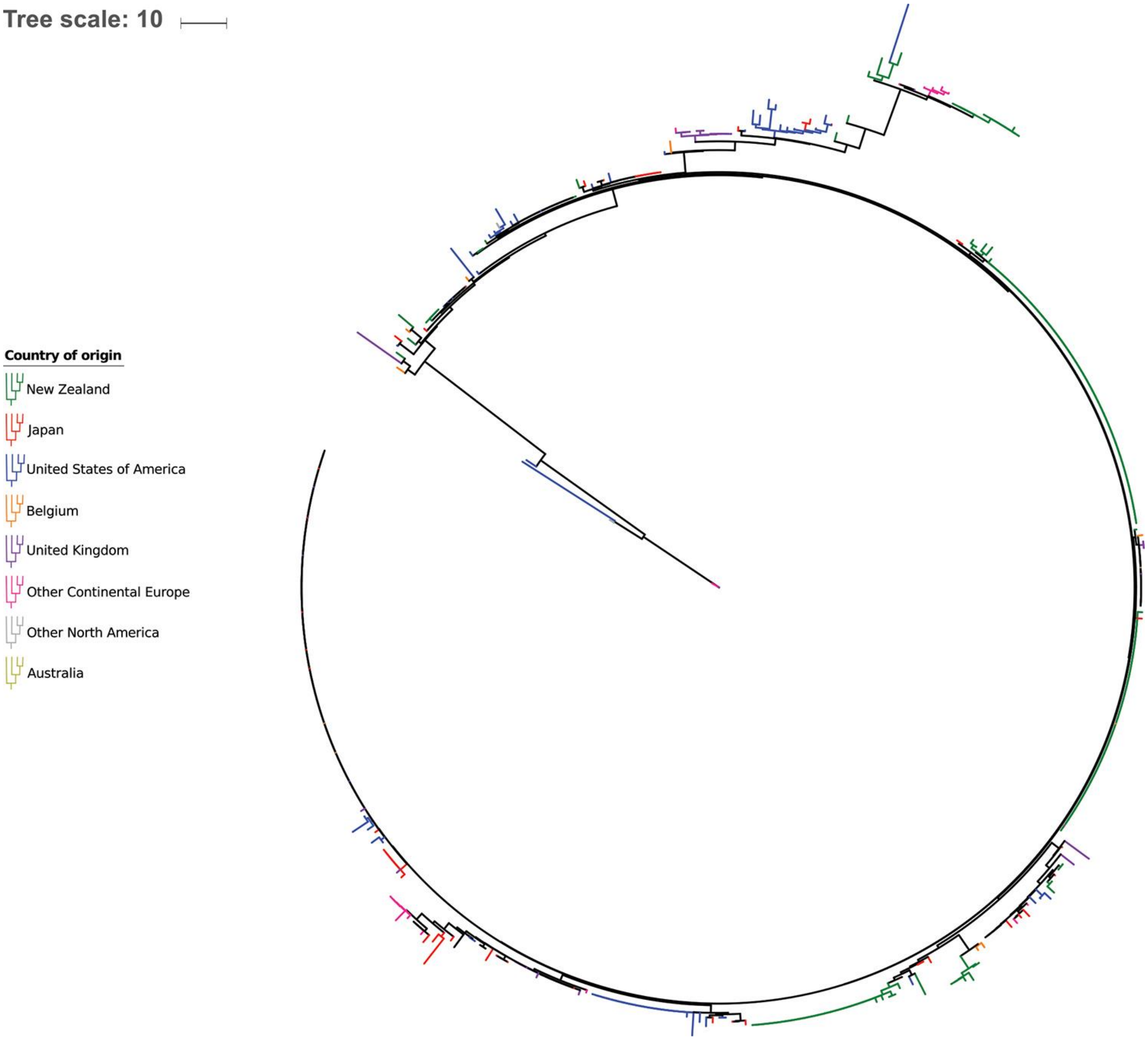

Appendix 1 Figure 2. Neighbor-joining tree of virulence genes (n = 192) of Escherichia coli serogroup O26 isolates (n = 404) with real branch lengths annotated by country.

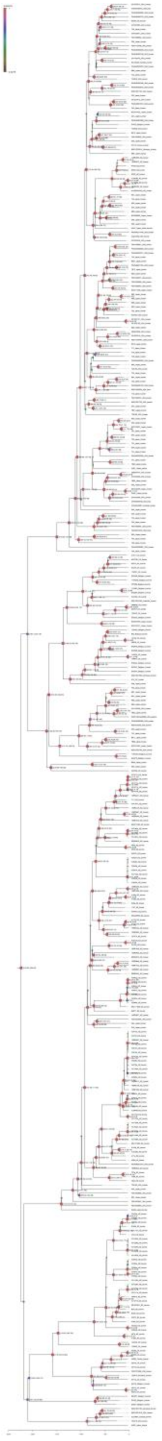

Appendix 1 Figure 3. Maximum clade credibility tree of time of most recent common ancestor (TMRCA) analysis of Escherichia coli serogroup O26 sequence type 21 (ST-21) isolates (n = 344), annotated with posterior probability of tree branches. All convergence dates are annotated with 95% HPD intervals from the age of the newest isolate (2017.5 in decimal years).

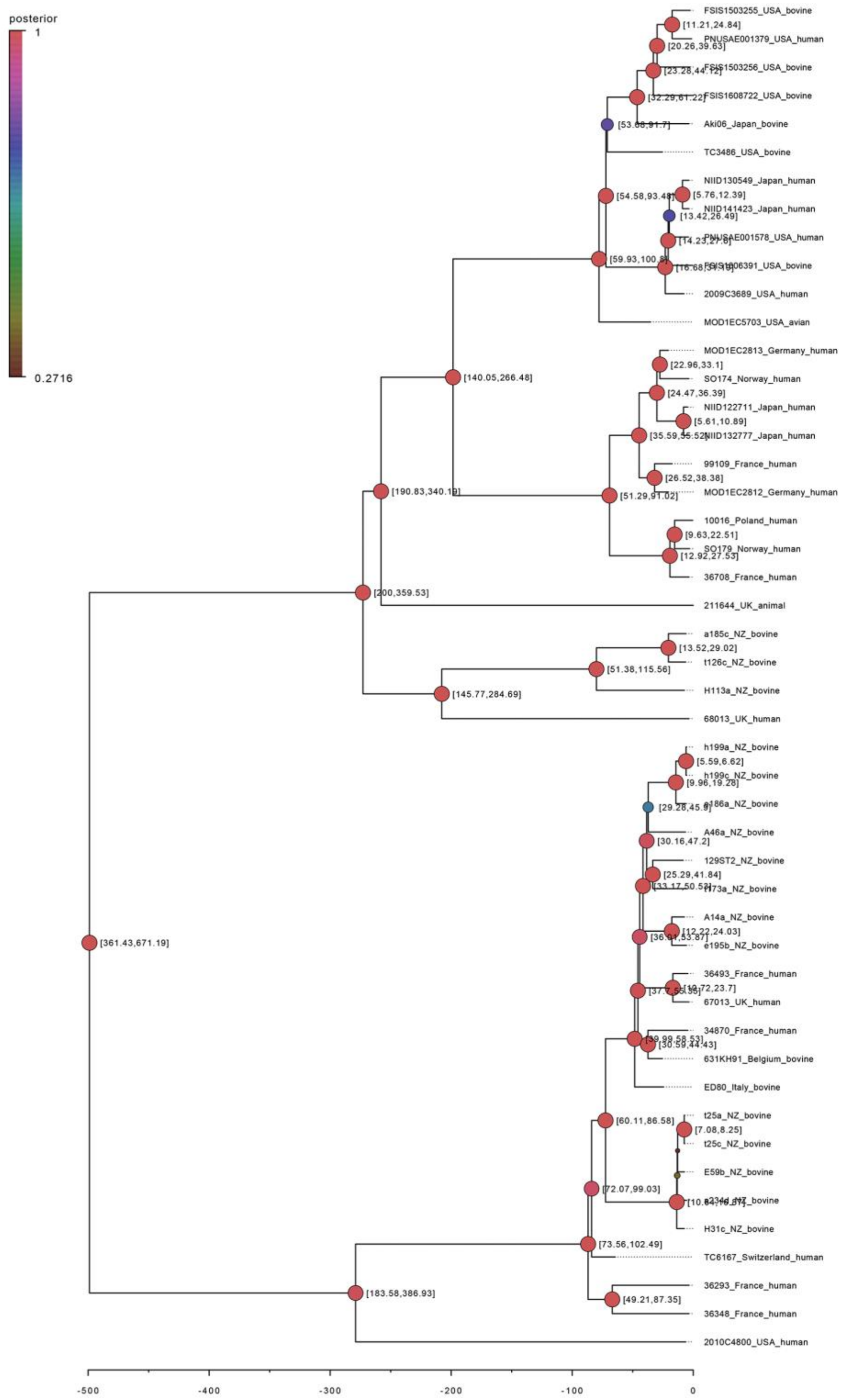

Appendix 1 Figure 4. Maximum clade credibility tree of time of most recent common ancestor (TMRCA) analysis of Escherichia coli serogroup O26 sequence type 29 (ST-29) isolates (n = 48), annotated with posterior probability of tree branches. All convergence dates are annotated with 95% HPD intervals from the age of the newest isolate (2017.0411 in decimal years).
